# Understanding the preferences of younger women for the delivery of a service to predict breast cancer risk: a discrete choice experiment

**DOI:** 10.1038/s44276-026-00209-x

**Published:** 2026-03-13

**Authors:** Stuart J. Wright, Shabnam Thapa, Amber Salisbury, Sarah Hindmarch, David P. French, Sacha J. Howell, Katherine Payne

**Affiliations:** 1 Manchester Centre for Health Economics, Division of Population Health, Health Services Research and Primary Care, School of Health Sc iences, Faculty of Biology, Medicine and Health, The University of Manchester, Manchester, UK; 2 National Institute for Health and Care Excellence, Manchester, UK; 3 Manchester Centre for Health Psychology, Division of Psychology and Mental Health, School of Health Sciences, Faculty of Biology, Medicine and Health, The University of Manchester, Manchester, UK; 4 Manchester Cancer Research Centre, Division of Cancer Sciences, School of Medical Sciences, Faculty of Biology, Medicine and Health, University of Manchester, Manchester, UK

## Abstract

**Background:**

This study aimed to understand the preferences of a sample of younger women (30–39 years) for the attributes of models of service delivery for a breast cancer risk-prediction service to identify how best to design a service to optimise uptake.

**Methods:**

A discrete choice experiment was used to quantify the preferences of a purposive sample of younger women (aged 30–39) without prior knowledge of their risk of developing breast cancer. Respondents chose from a series of questions including two unlabelled alternatives, representing different models of a risk-prediction service, and an opt-out alternative. Data were analysed using random parameter logit and latent class models to explore potential heterogeneity in preferences for the intervention.

**Results:**

The predicted uptake for a risk-prediction service ranged from 77 to 89%. Participants preferred a service with more flexible appointments which could be booked by the individual themselves. Latent class analysis suggested that around 7% of women would never have their risk predicted and for approximately 30% of women the choice would depend on the design of the service.

**Conclusion:**

Younger women would be likely to choose to have their breast cancer risk predicted, although some groups were sensitive to the design of the prediction service.

## Introduction

In the UK, breast cancer is the most common type of cancer among women with around 56,000 new cases diagnosed every year [1]. The NHS Breast Screening Programme invites women between 50 to 71 years every three years for breast screening [2]. Although, breast cancer is most commonly diagnosed in women aged 50 years or older, around 18% of cases are found in women under 50 years, and BC is the most common cause of death in women aged 35–49 years [1]. Women under 50 years can only get access to screening and preventative measures if they have a strong familial history of breast cancer conferring at least a 17% lifetime risk of the disease [3]. However, around two-thirds of women under the age of 50 years who develop breast cancer do not have any first or second degree family history at all [4]. Breast cancer in these younger women is more commonly lethal, due to and increased incidence of more aggressive subtypes and later stage presentation due to the lack of screening provision [5, 6]. The current surveillance strategy of relying on the presence of a strong family history may be inadequate because it fails to find the majority of younger women who are at increased risk of breast cancer. To address this gap the ‘Breast Cancer Risk Assessment in Young Women’ (BCAN-RAY) study (NCT05305963) has designed and evaluated a novel model of service delivery to offer risk assessment for breast cancer in women aged 30–39 years (hereafter ‘younger women’) [6].

There are a number of benefits to identifying younger women at higher risk of breast cancer. For these women, screening could be started at a younger age to catch cancers at an early stage. Alternatively, women could be provided with advice about lifestyle changes which could help to reduce their breast cancer risk or could be prescribed risk-reducing medicines [7, 8]. The potential benefits of such risk assessment to identify younger women at risk of breast cancer will only be realised if there is sufficient uptake of the service. The uptake of any service will be influenced by an individual’s preference for how the service is designed [9]. Before a model of service delivery has been rolled out it is clearly impossible to collect data on preferences for aspects of the model (revealed preferences) or uptake of the model. Stated preference methods, such as discrete choice experiments (DCEs), have a role when designing new models of service delivery [10]. A DCE asks a pre-defined group of relevant individuals (the sample) a series of choice questions in which they select their preferred option described using a set of attributes (the characteristics of the service delivery model) defined using levels (the possible range to define each characteristic) [11, 12]. The respondents’ choices are then analysed (using regression methods) to generate a measure of the samples’ preferences, which can be used to understand the relative importance of each attribute and the trade-offs between attributes [11, 13]. The outputs from the regression analysis can also be used to estimate the future uptake for exemplar models of service delivery [14].

This study aimed to understand the preferences of a sample of younger women (30-39 years) for the attributes of models of service delivery for a breast cancer risk assessment service. The study also aimed to generate estimates of the potential uptake of specified models of service delivery.

## Method

A discrete choice experiment, embed in an on-line survey, was designed to elicit the preferences of a sample of younger women for a model of service delivery for a breast cancer risk-prediction service. The DCE was designed and analysed following published methodological recommendations [15] and reported in line with a published checklist [16] (see Appendix 1). Ethical approval was obtained from The University of Manchester’s Proportionate Research Ethics Committee (reference: 2024-21125-37858).

### Conceptualising the choice question

To conceptualise the choice question, the integrated screening action model (I-SAM) of cancer screening behaviour was used as a framework for considering the steps needed for a woman to take part in a breast cancer risk-prediction service to guide decisions about early intervention such as receiving earlier breast screening [17]. This framework suggests that women have to go through multiple stages to take up the intervention on offer: becoming aware; becoming informed; deciding to act; acting; and repeating if necessarily. When considering a risk prediction service, the deciding to act and acting domains must be expanded to consider the stages of women making a decision to have their risk predicted, acting to have their risk predicted, deciding to receive their risk feedback, acting to receive their feedback, decided to act on their risk information to reduce their risk, actually acting to change their cancer risk.

As the BCAN-RAY study aimed to explore the feasibility of introducing a breast cancer risk-prediction service for younger women, this DCE focuses on women’s decision as to whether in principle they would like their risk to be predicted or not. It was decided that including questions to ascertain if women would then decide to receive their risk feedback and act on their risk information to reduce their risk (using strategies provided by the health service), would make a single survey too long to complete.

Firstly, for women to choose to receive risk-prediction, they must be aware of the service. As such, the sample to be recruited for this study was defined as women who would potentially receive the service: women between the ages of 30–39. Secondly, to decide to receive risk-prediction, women must be adequately informed about the service. As such, in the discrete choice experiment, information materials explaining the concepts of breast cancer risk-prediction were included at the start of the study. These were modelled as closely as possible on existing National Health Service leaflets for breast cancer screening [18].

### Survey design

The DCE was embedded into an online survey which was programmed in Qualtrics. Women were first shown a participant information sheet and asked to tick a box indicating they provided informed consent to taking part in the study. The survey (Appendix 2) comprised 5 sections: (i) an introduction to the survey explaining what is involved with risk-prediction for breast cancer in younger women (referred to as ‘training materials’ in a DCE); (ii) the choice questions; (iii) questions regarding respondents’ views on the survey; (iv) attitudinal questions about their risk behaviour and healthcare decision-making and (v) sociodemographic questions about themselves.

### DCE design

The DCE was framed around the choice question: “If you had to choose between the following breast cancer risk-prediction services, which would you choose?”. The respondents were asked to choose between two unlabelled (generic) alternatives and an opt-out option. The alternatives and opt-out option were described using six attributes assigned levels (see Table 1). The opt out option was described with fixed text: “You would not have your breast cancer risk predicted, you would be invited to breast cancer screening at age 50, if you were worried about cancer before this you would visit your GP”. An infographic was also included showing that 0 out of 100 people would be identified at high risk.

**Table 1:** Attributes and levels included in the DCE

Attribute	Description	Levels	Attribute Type (coding for analysis)
How risk is predicted	The combination of interventions used to predict a woman’s risk of breast cancer	• A questionnaire only • A questionnaire and mammographic breast density • A questionnaire and radiofrequency breast density • A questionnaire and genetic test • A questionnaire, mammographic breast density, and genetic test • A questionnaire, radiofrequency breast density, and genetic test	Categorical (Effects coded)
Appointments needed to predict risk	How many appointments would a woman need to attend to have her risk predicted	• One • Two	Categorical (Effects coded)
Location of appointment	Where the woman would need to go to have her risk predicted	• Home • General Practitioner (GP) • A mobile van • Hospital • Community Centre	Categorical (Effects coded)
Possible Times for the Appointment	Which days and what times of day appointments are available to book	• 9am-5pm weekdays • 9am-5pm weekdays and evenings or weekends	Categorical (Effects coded)
How appointments are booked	What the woman needs to do to book an appointment to have her risk predicted	• You are sent a litter with a fixed date and time • You can book a date and time yourself online or on the phone	Categorical (Effects coded)
The likelihood that you are predicted to be at high risk of breast cancer	The probability that the results suggest a woman should be classed as high risk and receive earlier interventions to reduce the risk of cancer or identify cancers at an earlier stage	• 5 out of every 100 (5%) people would be predicted to be high risk • 10 out of every 100 (10%) people would be predicted to be high risk • 15 out of every 100 (15%) people would be predicted to be high risk • 20 out of every 100 (20%) people would be predicted to be high risk	Continuous (Linear in main analysis; with checks for non-linear functional forms)

The attributes and levels for this study were identified using seven focus groups (with 29 women) and eight semi-structured interviews conducted online with women aged 30–39 years for a breast cancer risk assessment [19]. These semi-structured focus groups and interviews were designed with input from patient and public involvement. The qualitative data were used to generate a long list of 19 potential attributes. This long list was grouped into three categories: attributes of information about the risk-prediction service; attributes of the risk-prediction intervention itself; attributes of the process of returning risk information. The research team originally considered including two separate DCE tasks with the first focussing on women’s preferences for the actual risk prediction service and the second on women’s preferences for how risk information is returned. However, due to the length of the survey, it was considered that including two DCE tasks would potentially result in participants losing concentration over the survey. As such, it was decided among the research team to concentrate on the core attributes of risk prediction itself in this study. A final list of six attributes was produced by the research team (see Table 1). The research team focussed on defining attributes and levels that would describe a risk assessment service that was feasible to deliver.

### The experimental design

Experimental design for discrete choice experiments is the creation of choice questions by combining attributes and levels in a way which maximises the probability that preferences for all of the attributes and levels can be estimated with the lowest level of statistical uncertainty (statistical efficiency) [20]. A full factorial design would result in an unfeasible number of 921,600 potential combinations of attributes and levels in choice sets. A D-efficient, main effects design was created using the choiceDes package in the programming software R [21]. Illogical combinations of attributes and levels such as having a mammogram at home were removed from the design informed by expert clinical advice. The final experimental design comprised three blocks of ten questions with each participant randomised to receive one block. As 5 out of 6 attributes were categorical, a dominance test question was not included in the DCE design.

### Background questions

Background questions were included in the online survey to enable a description of the study sample and also for use when analysing for preference heterogeneity. The questions included were: sociodemographic questions including level of education, religion, ethnicity and whether they had children. Respondents were also asked about their attitude to risk and questions about their level of health information seeking or avoiding behaviour.

### Piloting

The survey was quantitatively piloted using a purposive sample of younger women (n = 50) adults recruited through an online panel-provider (Pureprofile). The results were then analysed using a conditional logit model to ensure that the coefficients for all attributes and levels could be estimated. The experimental design for the study was not updated using the results of the quantitative pilot.

### Study population and sample

The relevant study population was framed around younger women (aged between30 and 39 years) who by definition, all have an as yet (undefined) risk of developing breast cancer at some point in their lives. Participants who had previously been diagnosed with breast cancer or had a close relative with breast cancer were also excluded as individuals with a family history of cancer are already potential eligible for earlier interventions in the NHS. The online survey was fielded to a sample of younger women living in the UK recruited using an online panel-provider (Pureprofile). There are no acceptable statistical approaches to set the required sample size for a DCE. This study used the Orme rule of thumb to calculate a minimum sample size of 150 participants needed.

Although a sample size of 150 was the minimum required to estimate the preferences of the sample, a final target sample size of 1000 was set to allow for understanding heterogeneity in preferences. Respondents were sent a link to the online survey, and reminders were not used. Respondents who completed the survey in a time that was under 2 standard deviations from the median were defined as ‘speedsters’ and not engaging with the survey and removed from the dataset. These speedsters were then ‘replaced’ by a sample of further respondents identified by the panel-provider. Using Qualtrics also allowed the identification of responses which were likely from ‘bots’ completing the survey. These bots were ‘replaced’ by a sample of further respondents identified by the panel-provider.

### Data analysis

An analysis plan was created which specified that individuals who did not complete the survey and those who always chose the same alternative would be excluded. Speedsters and bots were replaced at the data collection phase. Descriptive statistics for sociodemographic characteristics, behavioural questions and survey feedback were produced for respondents in the final sample.

Following data cleaning, the choice data were analysed using conditional logit models in which the continuous attributes were specified as linear, continuous variables and categorial attributes effects coded. A single constant was included to represent the probability of opting in versus opting out.

Different model functional forms will be estimated whereby two constants are used to represent the probability of selecting hypothetical risk-prediction or feedback scenario A or scenario B. This serves as a test as to whether participants were always choosing scenario A or B regardless of the levels shown.

A series of regression models were then used to assess non-linearity in preferences for the probability of being identified as high risk attribute. All tests of model specification will be made by comparing the Bayesian Information Criterion (BIC) of the different models. If a model specification is found to result in a lower BIC value then this suggests that the model specification adds sufficient additional explanatory power for the number of additional parameters in the model.

When a final functional form was selected, a random parameter logit model was then estimated to determine if a model that allows for preference heterogeneity provided a better fit for the data. A two-step process was followed, with an uncorrelated random parameter logit estimated first and then a fully correlated random parameter logit estimated. The fully correlated model allows for both differences in error between participants as well as differences in preferences.

To better understand whether there were particular groups with similar preferences, a latent class model was also be estimated. The best number of classes was chosen using the BIC statistic. When the number of classes was chosen, a further model was estimated to determine if any demographic characteristics were correlated with membership of the classes. All of the collected demographic classes were tested for class membership prediction.

Coefficients and associated robust standard errors (SEs) from the best-fitting model were used to calculate predicted uptake probabilities for different hypothetical risk-prediction services. The hypothetical services reported in this paper are the most and least preferred services based on the choice model for aggregated preferences as well as an exemplar service representing the risk-prediction approach used in the BCAN-RAY study. Differences in predicted uptake among the different predicted classes from the latent class analysis will be explored.

All analyses were conducted using the Apollo package (version 0.3.5) in the open source software R [22, 23].

## Results

A sample of 936 younger women were included in the final analysis in this study. A total of 2512 woman entered the survey from the link sent by Pureprofile. Of these women, 1312 consented to take part, and 1144 of these completed the whole survey. The reCAPTCHA tool included in the survey identified 158 responses which were likely to have been provided by bots (with a score over 0.5). A further 28 responses removed due to fast completion times ( < 192 seconds: over 2 standard deviations from the mean). 22 respondents did not complete all the DCE questions and were excluded. In the final sample of 936 participants the median survey completion time was 9.38 min.

Descriptive statistics summarising the final sample are provided in Table 2. A summary of the results of the attitudinal questions is provided in Table 3.

**Table 2:** Demographic composition of the sample

Characteristic	Number (Percentage)
**Highest education**	
No formal education	13 (1.4)
1–4 O levels/GCSEs	50 (5.3)
5 + O levels/GCSEs	44 (4.7)
National Vocation Qualification (NVQs)	86 (9.2)
A levels/AS levels	148 (15.8)
Undergraduate degree	383 (40.9)
Postgraduate degree	175 (18.7)
PhD/Doctorate	15 (1.6)
Other formal qualifications	22 (2.4)
**Religion**	
No religion	479 (51.1)
Christian	354 (37.8)
Buddhist	8 (0.9)
Hindu	11 (1.2)
Jewish	1 (0.1)
Muslim	65 (6.9)
Sikh	5 (0.5)
Other	13 (1.4)
**Ethnicity**	
White English/Welsh/Scottish/Northern Irish/British	639 (68.2)
White Irish	10 (1.1)
White Gypsy or Traveller	2 (0.2)
Other white background	61 (6.5)
White and Black Caribbean	8 (0.8)
White and Black African	12 (1.3)
White and Asian	10 (1.1)
Other mixed/multiple backgrounds	7 (0.7)
Indian	26 (2.7)
Pakistani	19 (2.0)
Bangladeshi	11 (1.2)
Chinese	11 (1.2)
Other Asian Background	18 (1.9)
Black African	79 (8.4)
Black Caribbean	14 (1.5)
Any other Black/African\Caribbean Background	3 (0.3)
Arab	2 (0.2)
Any other ethnic group	4 (0.4)
**Do you have any children?**	
Yes	572 (61.1)
No	364 (38.9)

**Table 3:** Summary of responses to attitudinal questions

Risk preferences	
Overall level of risk taking (from 0 for risk averse to 10 for fully prepared to take risk)	5.41 (CI 5.24–5.58)
Willingness to take risks when driving	3.34 (CI 3.16–3.52)
Willingness to take risks in financial matters	4.44 (CI 4.26–4.62)
Willingness to take risks during leisure and sport	5.58 (CI 5.41–5.74)
Willingness to take risks in your occupation	5.20 (CI 5.03–5.38)
Willingness to take risks with your health	3.70 (CI 3.52 to 3.89)
Willingness to take risks in your faith in other people	4.94 (CI 4.77–5.11)
**Information Engagement (from 0 for not at all true for me to 4 for very much true for me)**	
I like to gather as much information as I can before making a decision	3.15 (CI 3.09–3.22)
I like to review information multiple times before making a decision	2.97 (CI 2.91–3.02)
After I’ve made a decision, I continue to look for related information	2.90 (CI 2.84–2.95)
I like to make decisions quickly (reverse scored when creating overall score)	1.97 (CI 1.90–2.05)
Mean Information Engagement	2.76 (CI 2.72–2.80)
**Information Apprehension (from 0 for not at all true for me to 4 for very much true for me)**	
I have difficulty making sense of information from multiple sources	1.80 (CI 1.72–1.87)
I fear that I might find out something that I don’t want to know	2.24 (CI 2.17–2.32)
I think it’s the doctor’s job to deal with information, not mine	1.54 (CI 1.47–1.61)
I feel overwhelmed by the amount of information available	2.20 (CI 2.13–2.27)
Mean information apprehension	1.94 (1.89– 2.00)

The average age of respondents in the final survey was 34.63 with an interquartile range of 5. Most participants were of white ethnicity (76%) and of no religion (51.1%) or Christian (37.8%). 61.1% of women had children. As a narrow age group was used for this study, statistics were not available to determine how representative the sample was of the UK population of women aged 30 to 39.

On average the participants stated that they were slightly more likely than average to take risks, although they were less likely to take risks with their health. Women in the sample tended to prefer to engage with information but had only average levels of information apprehension. However, the participants were more likely than average to agree with the statement “I fear that I might find out something that I don’t want to know” which may be particularly relevant when considering the concept of breast cancer risk-prediction.

On average the participants found the survey easy to complete (mean 3.87 out of 5). 54.6% of participants stated that they always used all of the attributes to make their decisions, 42.0% used a sub-set of attributes, and 3.4% said they never chose the risk-prediction service.

### Preferences

The results of the model selection process suggested that a model with a single constant for the opt in options was superior (BIC: 18187) to having separate constants for each opt in option (BIC: 18194). This suggested that there was no evidence that participants disproportionately chose either the left or right hand options in the choice tasks. In addition, no evidence was found of non-linearity in the likelihood of being predicted to be high risk attribute and as such a single linear coefficient was used for this attribute.

Different model specifications were explored to allow for preference and scale heterogeneity in the responses. The model fit statistics are available in supplementary appendix 2.2. The best model was an uncorrelated random parameter logit with pseudo panel effects. This model allows for differences in preferences among individuals as well as differences in error in completing the survey. The coefficients for this model are presented in Table 4:

**Table 4:** Model coefficients

Attribute or Level	Estimate	Standard Error	*P*-value
Number of appointments	−0.081	0.054	0.068
Appointments available at evenings and weekends	0.213***	0.025	< 0.001
Appointments only available during work hours	−0.213***	0.025	< 0.001
You can book the appointment yourself	0.141***	0.023	< 0.001
An appointment is booked for you	−0.141***	0.023	< 0.001
Location			
Hospital	−0.254***	0.053	< 0.001
Community Centre	−0.012	0.066	0.425
Mobile Van	0.008	0.054	0.443
Home	0.312**	0.128	0.008
General Practitioner	−0.052	0.054	0.328
Probability of being predicted to be at high risk	0.028***	0.006	< 0.001
Mode of risk-prediction			
Questionnaire only	−0.829***	0.069	< 0.001
Questionnaire and genetic test	0.127**	0.048	0.004
Questionnaire and mammography	0.071	0.067	0.146
Questionnaire, mammography, and genetic test	0.465***	0.069	< 0.001
Questionnaire and radiofrequency scan	−0.186***	0.045	< 0.001
Questionnaire, radiofrequency scan and genetic test	0.353***	0.059	< 0.001
Alternative specific constant^a^	3.993***	0.230	< 0.001
Sigma for the Panel Effect^b^	-0.283	0.053	< 0.001

^a^Representing the likelihood an individual would choose a risk-prediction service with mean effect for location, mode of risk-prediction, how the appointment is booked, and whether you can book yourself compared to no risk-prediction service.

^b^This coefficient represents the correlation of error in an individual’s responses across the multiple choice sets they answer

**Significant at the <0.01 level

***Significant at the <0.001 level

The results of the random parameter logit model suggest that the participants in this study were likely to choose to have their risk predicted, as shown by the large constant term. Participants valued a service that was more likely to identify women at higher risk. They were more likely to choose a service which was available in the evenings or weekends and could be booked themselves. Participants did not want to have to go to a hospital for risk assessment but were more likely to choose a service available at home. Participants were less likely to choose a risk-prediction service that only used a questionnaire to assess risk or used a questionnaire and radiofrequency scan. However, participants were more likely to choose a risk-prediction service with a genetic component to risk-prediction.

#### Latent class analysis

In the latent class analysis it was found that a model with four classes minimised the BIC, providing the most explanatory power for the number of parameters included. No demographic or attitudinal parameters were found to adequately predict class membership based on BIC, although the level of information apprehension did reduce the Akaike Information Criterion. As such, only a constant term was included to explain class membership.

The results of the latent class analysis are reported in Table 5. Nearly 60% of the sample belonged to class 1 which had strong preferences for a risk-prediction service. The preferences of this class were broadly similar to those of the aggregate sample, although they were also likely to attend a risk-prediction service provided in a mobile van. Class 2 comprised 18.4% of the sample and did not have strong preferences for any of the attributes and levels apart from the constant and adding a genetic test to questionnaire-based assessment. They also appeared to be sensitive to the number of appointments needed, although this was not statistically significant (p = 0.07). They were potentially a group who answered the survey in a random manner.

**Table 5:** Results of the Latent Class Analysis

	Class 1 (59.3%)		Class 2 (18.4%)		Class 3 (14.9%)		Class 4 (7.4%)	
Attribute or Level	Coefficient	*P*- value	Coefficient	*P*- value	Coefficient	*P*- value	Coefficient	*P*- value
**Number of appointments**	−0.016	0.800	−0.168	0.070	0.02	0.917	−1.18**	0.013
**Appointments available at evenings and weekends**	0.194***	0.000	−0.002	0.974	0.529***	0.000	0.183	0.316
**Appointments only available during work hours**	−0.194***	0.000	0.002	0.974	−0.529***	0.000	−0.183	0.316
**You can book the appointment yourself**	0.13***	0.000	0.015	0.715	0.276*	0.013	0.037	0.854
**An appointment is booked for you**	−0.13***	0.000	−0.015	0.715	−0.276*	0.013	-0.037	0.854
**Location**								
Hospital	−0.209**	0.004	−0.058	0.510	−0.259	0.366	0.029	0.932
Community Centre	0.004	0.962	−0.011	0.909	0.152	0.724	0.412	0.263
Mobile Van	0.196**	0.004	0.073	0.426	−0.589*	0.041	−0.098	0.814
Home	−0.055	0.802	0.035	0.810	1.017	0.352	0.111	0.818
General Practitioner	0.064	0.425	−0.039	0.643	−0.321	0.333	−0.454	0.255
**Probability of being predicted to be at high risk**	−0.002	0.764	−0.014	0.069	0.247***	0.000	0.028	0.400
**Mode of risk-prediction**								
Questionnaire only	−0.995***	0.000	0.071	0.487	0.225	0.456	−0.555	0.249
Questionnaire and genetic test	0.008	0.889	0.318***	0.000	0.105	0.515	0.159	0.710
Questionnaire and mammography	0.037	0.633	0.075	0.498	0.277	0.436	0.286	0.544
Questionnaire, mammography and genetic test	0.647***	0.000	−0.173	0.146	−0.169	0.559	0.212	0.660
Questionnaire and radiofrequency scan	−0.196***	0.000	−0.157	0.098	−0.216	0.210	−0.265	0.581
Questionnaire, radiofrequency scan, and genetic test	0.499***	0.000	−0.135	0.250	−0.223	0.230	0.163	0.741
**Alternative specific constant**	4.175***	0.000	0.528**	0.003	0.912	0.092	−2.202**	0.002
**Class Membership**								
**Constant**	Reference		−1.171***	0.000	−1.378***	0.000	−2.087***	0.000

**Significant at the <0.01 level

***Significant at the <0.001 level

People in class 3 (14.9%) of the sample were the only group without a significant alternative specific constant suggesting that they were more concerned with how a risk-prediction service was delivered than the other classes. They preferred appointments which were available at evenings and weekends and being able to book appointments themselves. They were averse to attending appointments at a mobile van and had a strong preference for a service which found more women at higher risk. Class 4 (7.4%) appeared to be unlikely to ever use a risk-prediction service, as demonstrated by their negative alternative-specific constant. This may also be supported by their dislike of services with more appointments as no risk-prediction service involves no appointments.

#### Uptake for a breast cancer risk-prediction service

Table 6 presents the predicted uptake for the most and least preferred breast cancer risk-prediction services and a service provided in a way similar to that in the BCAN-RAY study. Uptake was predicted using the random parameter logit model with pseudo panel effects and the latent class analysis, with uptake presented for each class and aggregated. For the full sample, both in the RPL and latent class analysis, predicted uptake for a breast cancer risk-prediction service is high regardless of the composition of the service (77% to 89%). In the latent class analysis it can be seen that class 1 virtually always choose to have their risk predicted while uptake for the BCAN-RAY and least preferred services are marginally lower in class 2 and class 3. The predicted uptake is more variable in class 3 who have different preferences for the attributes and levels to the other classes. This is driven by their dislike for the mobile van used in the overall optimal service and their increased willingness to use the questionnaire in the risk-prediction service which is otherwise least preferred.

**Table 6:** Predicted uptake for different breast cancer risk-prediction services using different models

	Random Parameter Logit	Latent Class Analysis				
Risk-prediction Service	Total	Class 1 (59.3%)	Class 2 (18.4%)	Class 3 (14.9%)	Class 4 (7.4%)	Total^a^
**Best**^**b**^	89%	100%	100%	66%	14%	87%
**BCAN-RAY**^**c**^	85%	99%	99%	63%	11%	86%
**Worst**^**d**^	77%	97%	90%	73%	4%	84%

^a^Total predicted uptake based on a weighted average of the uptake of each individual class

^b^One appointment, available evenings and weekends, can book yourself, in a mobile van, with a questionnaire, mammography, and genetic test, 20% predicted to be at high risk

^c^One appointment, available weekdays only, appointment booked for you, in a hospital, with a questionnaire, mammography, and genetic test, 20% predicted to be at high risk

^d^One appointment, available weekdays only, appointment booked for you, in a hospital, with a questionnaire only, 5% predicted to be at high risk

## Discussion

This discrete choice experiment has demonstrated that there would be significant demand for a breast cancer risk-prediction service among younger women if this were provided by the NHS. Uptake for an optimised risk-prediction service could be as high as 89%, with the worst potential service in this DCE still predicted to have uptake of 77%. Evidence provided by the latent class analysis demonstrates that while most women would attend a breast cancer risk-prediction service regardless of its design, around 7% of women would never want to have their risk predicted. In addition, the decision of around 30% of women in classes 2 and 3 to attend the service would be sensitive to the design of the service, with those in class 3 less likely to attend services which the majority of women find preferrable. This suggests the potential need to tailor services to different groups.

To date, the majority of research around breast cancer risk-prediction has focussed on its use to stratify screening intervals by risk. In such studies, risk assessment and interval stratification had “high, but not universal, acceptability” [24]. For example, in a cross-sectional survey of women aged 40–70 in England, Ghanouni et al found that 85% of women thought breast cancer risk assessment was a good idea while 74% were willing to have it [25]. These results are similar to the predicted uptake of 77–89% in this study.

While risk-prediction at the age of population screening may be acceptable for women, there may be additional barriers to risk-prediction in younger women compared to in its use for population screening. For example, risk-prediction for stratified screening is likely to be conducted at the first screening appointment so would not need additional visits. Similarly, breast density measurement can be conducted using the mammogram images taken as part of the woman’s first screen. A risk-prediction service for women attending at a younger age would require them attending a stand-alone appointment for risk-prediction unless this could be incorporated into another service such as cervical screening which currently invites women from the age of 25 in the UK. If a mammogram was required to measure breast density then this would likely involve having to attend an appointment at a hospital or mobile van. These factors mean that women offered risk-prediction at a younger age may face additional barriers to attending compared to women invited for risk-prediction at screening age. This study suggested that for most women, the need for additional appointments would not deter them from engaging with a breast cancer risk prediction service in primary care.

This discrete choice experiment suggested that for some women, other barriers may impact their decision as to whether to attend or not. Flexibility about appointment booking and availability of appointments were important factors in women’s choices about risk-prediction and women were averse to having to go to a hospital for risk-prediction. While women valued a service they could participate in from home, they disliked only completing a questionnaire and risk-prediction services with fewer women predicted to be at higher risk potentially offsetting the value of a home-based service. While radiofrequency-based breast density assessment may offer flexibility about the location of breast density assessment compared to mammography, as well as reduced risk of radiation exposure, it seemed to be less preferred by women. Further research is needed to understand women’s aversion to this technology. Potential explanations may include unfamiliarity with the technology compared to mammography or the potential mistaken belief that a low-dose mammography scan could identify cancers at the risk prediction appointment.

This discrete choice experiment found that women appeared to place a higher value on services with a genetic testing component included in risk-prediction. This effect is independent on any increase in the ability of the service to find women at higher risk of cancer despite the known clinical utility of genetic testing in breast cancer risk-prediction in practice. Previous discrete choice experiments have also found that people value genetic testing when the clinical utility of this is predicted to be low [26, 27]. Further research is needed to understand why women were more likely to choose genetics-based risk-prediction service. Possible explanations could be the additional benefit to a woman’s family of identifying particular genetic variations such as in the BRCA 1 and 2 genes. Alternatively, higher awareness in the influence of such genes on cancer risk than factors such as breast density may have influenced women’s choices. Another explanation may lie in the concept of genetic essentialism whereby individuals believe that it is genetics that fundamentally determine our health and outcomes in life and not other factors such as the environment [28].

There were a number of limitations to this study. Firstly, while attempts were made to recruit a representative sample of UK women for the study, the use of an online-only survey means that potential participants without a device that could access the internet were excluded. This means the survey is unlikely to be truly representative and may have excluded some women in lower sociodemographic groups. In addition, while the survey was representative in terms of the proportion of individuals of different ethnicities recruited, the small sample size of individuals from each ethnicity limits the ability to observe differences in preferences between groups. As such, further research is needed regarding preferences for breast cancer risk-prediction among different groups who struggle to access the health system. Over-sampling of women from specific groups or the use of different recruitment approaches may be required to recruit women from these groups.

This discrete choice experiment also concentrated solely on women’s preferences for risk prediction aspect of the risk prediction service. As identified when using the I-SAM model, there are multiple stages through which women must pass through to make a decision about participating in a risk prediction service. Once a woman has her risk predicted, she and her clinicians must then decide to act on that risk information, for example in engaging with early screening or by taking risk-reducing medication. Presenting risk information in an understandable format that meets women’s preferences is therefore essential to ensure that the potential clinical benefits of risk prediction are realised. This was not considered in this discrete choice experiment but should be explored in future research.

An additional limitation of this survey was potentially in the phrasing of the attribute “The likelihood that you are predicted to be at high risk of breast cancer”. This attribute aimed to represent the additional numbers of women who would be identified to be at high risk through using more intensive risk prediction strategies. The research team tried different ways of phrasing this in piloting, including focusing on the predictive ability of the risk prediction approach. However, this phrasing was deemed to be too complex. While the final chosen wording about the likelihood of being predicted to be at high risk of cancer could potentially be open to the interpretation that smaller numbers are better because you are then at lower risk, it was decided that this language was the best compromise. The significant, positive coefficient for the attribute in the random parameter logit model suggests that women generally interpreted this attribute in line with its true meaning. While women in latent class group 1 showed now significant preference for this attribute, this group were overwhelming positive about risk prediction in general so may simply have not used the quality of risk prediction to make their decisions. Further qualitative research could be beneficial in supporting researchers to understand how women trade off between more intensive risk prediction strategies and improved risk prediction performance.

While strategies were enacted to ensure the validity of responses, including the use of bot detection questions and filtering by completion speed, some responders may not have completed the survey in a manner that reflected their true preferences. While the chosen models allow for preference and scale heterogeneity, care must be taken when interpreting the results for the latent class analysis as groups may differ on either their preferences or error variance. In particular, participants in class 2 only have statistically significant preferences for the constant and using a questionnaire with genetic test. It may be that this group were quite random in their responses so care may be required when interpreting the results for this group.

## Conclusion

This study suggests that there would be strong demand from women between the ages of 30 and 39 for a service to predict their risk of breast cancer. While most women would want their risk predicted regardless of the design of the service, the choices of a minority would depend on how the service is offered by the health system. Consideration should be given to making services accessible to all to realise the benefits of the service in reducing the number of cancers in this age group or in finding cancers at an earlier stage.

## Supplementary information

Supplementary Appendix 1

Supplementary Appendix 2

## Data Availability

The survey used to collate the data for this study is made available in supplementary appendix 1 as a pdf version of the online survey. The data generated by the survey has been made publicly available with informed consent from the research participants via figshare. Participants gave their informed consent to the publishing of the data. No personal information is contained in the data: https://figshare.com/articles/dataset/BCAN-RAY_DCE_raw_data/29597318?file=56376407, https://figshare.com/articles/dataset/BCAN-RAY_DCE_cleaned_data_for_analysis/29597330?file=56376617.
