# Supplementary material for: Understanding the preferences of younger women for the delivery of a service to predict breast cancer risk: a discrete choice experiment
Source: BJC Rep. 2026 Mar 13;4:10. doi: 10.1038/s44276-026-00209-x (PMC12987935; doi:10.1038/s44276-026-00209-x)
Supplement: Supplementary file 1 — Supplementary Appendix 1 [file 44276_2026_209_MOESM1_ESM.pdf]

The University of Manchester

Welcome

Understanding Women's Preferences for Breast Cancer Risk Prediction

Thank you for your interest in completing this survey. In this survey we'll show you some information about the importance of understanding a women's risk of developing breast cancer. We will also explain how it is possible to predict the risk of younger women developing breast cancer in the future.

We will ask you some questions about what you think about a new service to predict the risk of younger women developing breast cancer in the future. We are interested in hearing everyone's views, whether they think they would like to have their breast cancer risk predicted or not.

In this survey we will not predict your risk of breast cancer and will not collect information which would allow us to do this. We will not collect any information which could be used to identify you

There are 4 sections in the survey
1.Background information to allow you to complete the survey
2. Your views on a service to predict the risk of breast cancer in young women
3. Your thoughts on the questions
4. Some general questions about you.

We recommend completing this survey on a computer for the best user experience. It will still work on a mobile phone but may not display as well

Eligibility

We first need to ask some questions to understand if you are eligible to complete this survey

What is your age?

What is your sex?
A question about gender identity will follow later on in the

questionnaire

- Male
- Female

Is the gender you identify with the same as your sex registered at birth?
This question is voluntary

- Yes
- No

Have you previously been diagnosed with breast cancer or are you currently receiving care from a breast cancer family history clinic because you have been assessed to be at higher risk of breast cancer?

- Yes
- No

Have any of your close relatives ever been diagnosed with breast cancer? Examples of close relatives may include your mother, sisters, grandmother, aunts, or male relatives who have had breast cancer

○ Yes

○ No

pis

Exploring the Preferences of Young Women for a Breast Cancer Risk Prediction Service

Participant Information Sheet (PIS)

You are being invited to take part in an online survey about your preferences for a potential breast cancer risk prediction service. Before you decide whether to take part, it is important for you to understand why the research is being conducted and what it will involve. Please take time to read the following information carefully before deciding whether to take part, and discuss it with others if you wish. Please ask if there is anything that is not clear or if you would like more information. Thank you for taking the time to read this.

About the research

Who will conduct the research?
This research is being conducted by Dr Stuart Wright, Manchester Centre for Health Economics, The Division of Population Health, Health Services Research and Primary Care, The University of Manchester

What is the purpose of the research?
In the UK, breast cancer screening is offered to women between the ages of 50 and 70. However, 20% of breast cancers occur in women under the age of 50. There are many things which affect a woman's risk of breast cancer, including family history of breast cancer, whether she has had children, the density of her breasts, and genetics. A healthcare service which offered women the opportunity to have their risk predicted may help to reduce the number of cancers in younger women by allowing those at high risk to take medicine to reduce their cancer risk, or to come to breast screening from an earlier age. This study aims to explore whether women would be interested in having their risk of breast cancer predicted and how such a service should be designed.

You have been invited to take part as you are a woman between the ages of 30 and 39, who does not have a close family history of breast cancer. This means you would potentially be invited to such a service if it existed in the National Health Service. We are interested in your views, whether you think you would be interested in having your risk of breast cancer predicted or not.

Who has reviewed the research project?
This project has been reviewed by The University of Manchester Proportionate Research Ethics Committee.

Who is funding the research project?
This research is part of the Breast CANcer Risk Assessment in Younger Women (BCAN-RAY) study which is funded by Cancer Research UK.

What would my involvement be?

What would I be asked to do if I took part? You would be asked to complete an anonymous online survey hosted on Qualtrics.
You will be asked to complete an online survey with 5 parts. In the first part of the survey you will be shown some information about breast cancer risk and the potential benefits of predicting women's risk. You will then be shown a series of questions where you will be presented with hypothetical (made up) risk-prediction services which vary in different ways. In each question you will be asked to choose which risk-prediction service you would prefer or whether you would prefer not to have your risk of breast cancer predicted. You will then be asked to complete the same type of exercise about how you would want your risk information provided to you. You will then be asked some questions about how you found completing these questions. Finally, we'll ask you some questions about you. These questions won't ask for any personal information. The survey should take around 20 minutes to complete.
Please note, the survey will not collect your IP address.

Will I be compensated for taking part?
You will be provided with compensation for completing the survey by PureProfile (the market research agency supporting this survey).

What happens if I do not want to take part or if I change my mind?
It is up to you to decide whether or not to take part. If you do decide to take part you will be given this information sheet to keep and will be asked to tick a box to confirm consent. If you decide to take part you are still free to withdraw at any time without giving a reason and without detriment to yourself up until the point that you submit your online responses. It will not be possible to remove your data from the project once you have submitted your responses as they are collected anonymously and we will not be able to identify your specific data. This does not affect your data protection rights. If you decide not to take part you do not need to do anything further.

For detailed information about how we plan to use and store the information that you share with us, please read our **Data Protection, Confidentiality and Further Details page at the following link**

Data Protection, Confidentiality and Further Details

What should I do if the survey makes me feel upset, worried, or distressed?
If the survey makes you feel upset, distressed, or worried about your risk of breast cancer there are organisations who may be able to help. Macmillian Cancer Support offer a range of support including information and a telephone support line which can be reached at 0808 808 00 00. You can also email them or chat online. Further details are available at:

https://www.macmillan.org.uk/cancer-information-and-support/get-help
You may also wish to discuss your worries with your GP.

Contact Details

If you have any queries about the study or if you are interested in taking part then please contact the researcher(s): DR STUART WRIGHT. Email: stuart.j.wright@manchester.ac.uk

-

I confirm that I have read and understand the information about this study

- Yes
- No

Do you consent to taking part in this study?

- Yes
- No

Captcha

Thank you for agreeing to take part in this study. Please tick the box below to continue.

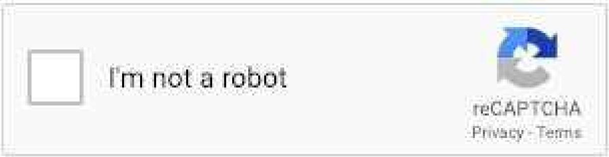

Information

Thank you for agreeing to take part in this study

We will now show you some information about a potential service to predict the risk of breast cancer in young women. After you have looked at this information we will ask you some questions about your preferences for a service to predict the risk of breast cancer in younger women. We are interested in your views whether you would be interested in such a service or not.

What is Breast Cancer?

Breast cancer is the most common type of cancer in the UK. Around 55,000 women are diagnosed with breast cancer each year in the UK. 1 in 7 women will develop breast cancer in their lifetime.

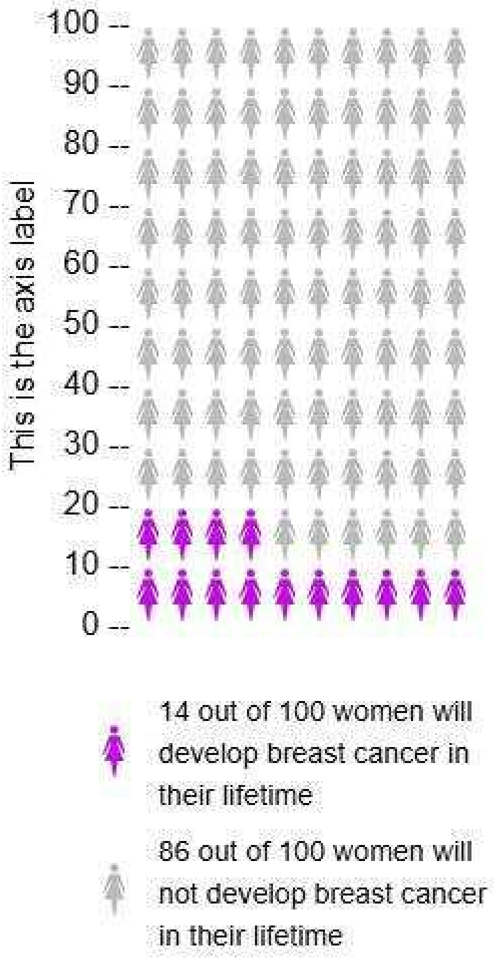

Breast cancer occurs when cells in the breast grow in an uncontrolled way and build up to form a tumour. As the tumour grows, cells can eventually spread to other parts of the body and become life-threatening.

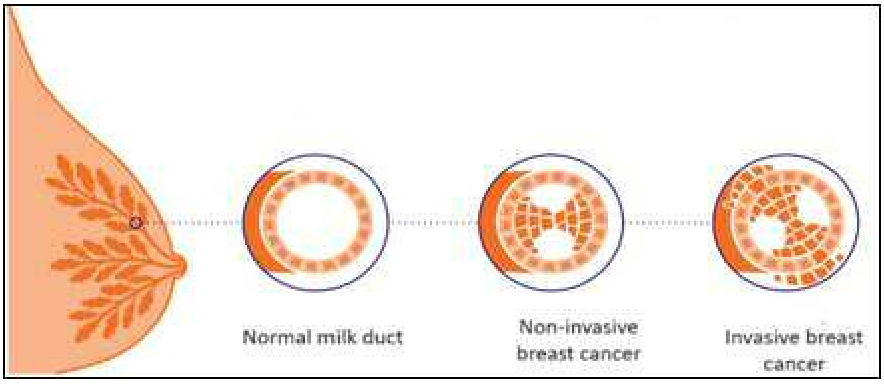

The risk of getting breast cancer goes up as you get older. Most cases of breast cancer are found in women over 50 years.

This figure shows that out of 100 cases of breast cancer, 80 will be in women over 50 years of age.

The NHS Breast Cancer Screening Programme

The NHS Breast Screening Programme invites women between 50 to 71 years every three years for breast screening. During breast screening, two X-rays (mammograms) of each breast are taken. The mammogram checks the breasts for any signs of cancer and can spot cancers that are too small to see or feel. The picture below shows how a mammogram is taken.

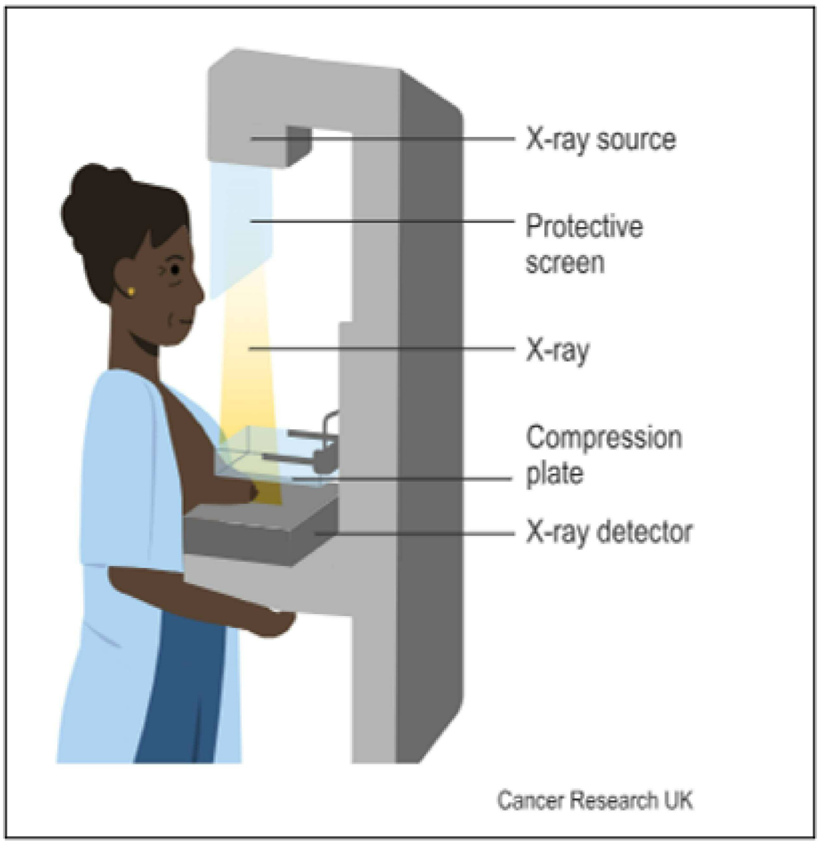

Screening finds breast cancer at an early stage when cancers can be more easily treated. But screening does not prevent you from getting breast cancer.

Breast screening does have some risks.

- There is a chance that screening might miss cancers.
- Screening can also find cancers which are not aggressive and may never have grown to a dangerous size. Finding these cancers means that they have to be treated even though they wouldn't have caused any health problems.
- Some women find the mammogram uncomfortable or painful

Currently there is no programme to screen for younger women for breast cancer

Regular breast screening is not recommended for **all** women under the age of 50, as in this age group the harms outweigh the benefits.

**Some examples of harms from offering breast screening to women under age of 50-years**
Longer term radiation exposure from x-rays which may cause cancer
Higher risk of being told you might have cancer when you don't (a false-positive)
Higher risk of finding very slow growing cancer which might not have caused any harm

Only women under the age of 50 who have a family history of breast cancer can get access to regular breast screening and preventative care (like being prescribed medicine to reduce their risk of cancer). This is because their risk of breast cancer is higher than the average woman of their age.

Breast Cancer in Younger Women

Although for younger women the harms of screening outweigh the benefits, identifying breast cancers in this group is very important because:

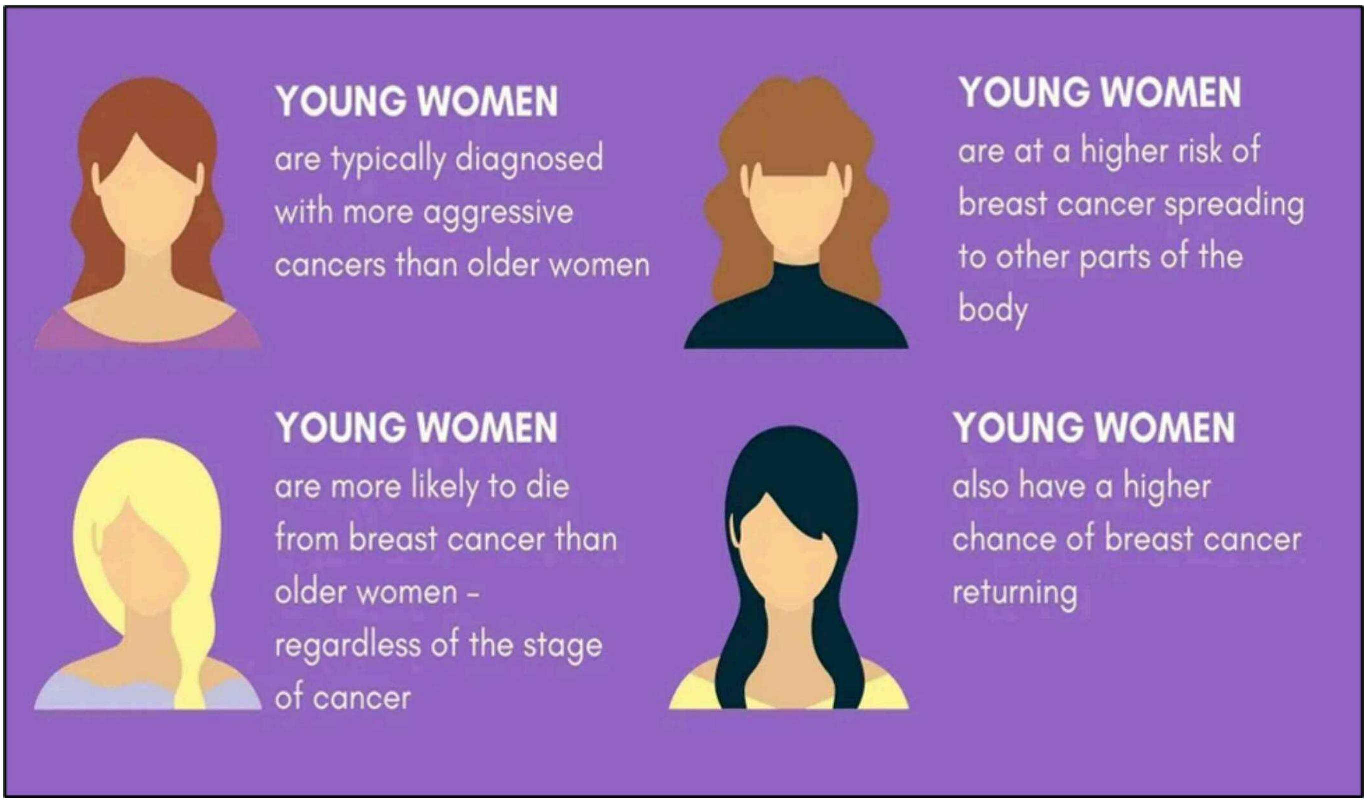

Currently the only way that cancers can be found in most younger women is when they find a lump through self-breast checking and contact their GP.

Rather than offering ALL women under 50-years of age an invitation to breast screening, we are interested in an approach that cab be used to predict their risk of developing cancer in the future. Then women who are more likely to develop cancer (called 'high-risk') can be targeted and offered

breast screening

Proposed New Risk Prediction Service

Research is currently being carried out to find a good way of predicting younger women’s risk of developing breast cancer in the future. If an accurate way of predicting risk of breast cancer is found, this could be offered in the NHS.

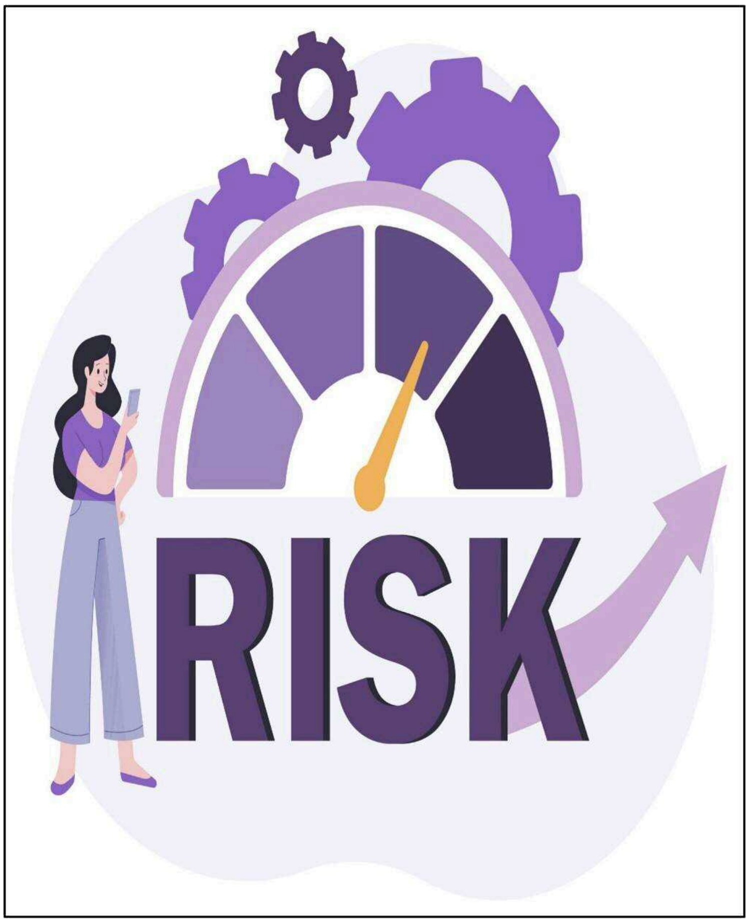

This potential new service would offer women the opportunity of knowing their risk of developing breast

cancer in the future

Women found to be at a higher than normal level of risk could be offered:

- breast screening to catch cancers
- advice on reducing their risk through diet and exercise
- medication to reduce their risk of developing cancer in the future.

Results from a Breast Cancer Risk Prediction Service

A breast cancer risk prediction service would predict whether you are at increased risk or not at increased risk of developing breast cancer. For women between the ages of 30 and 39 the average risk of developing breast cancer in the next 10 years is about 0.5% (5 in 1000 women).

Women found to have a 3% or higher risk of developing breast cancer over the next 10-years are considered to be at **high** risk of developing cancer. This means that there is an over 3 in 100 chance that you will develop breast cancer within the next ten years.

Women with a 10-year risk of breast cancer lower then 3%

(3 in 100) are not considered to be at higher risk of breast cancer. This means your risk is same as the average population level.

If you are found to be at increased risk:

- You might be able to access breast cancer screening before the age of 50 so that if you develop cancer in future, it is caught at an early stage.
- You will also be able to access medicine that decreases your risk of getting breast cancer. Studies have shown that these medicines can reduce the risk of develping cancer form between 30 and 40%.
- You may also be provided with lifestyle related advice, such as information about diet and exercise to help to reduce the overall risk of developing breast cancer.

If you are not at increased risk:

- You will be provided with information on breast awareness and health related behaviour so that you can prevent your risk from increasing in the future.
- You will be invited for breast cancer screening when you reach 50 years of age.

Potential Harms of a Risk Prediction Service

A risk prediction service could be a good way to make yourself aware about your risk status for breast cancer. It would allow you to take steps to lower your risk or catch cancer at an earlier stage. However, there are some potential harms as well:

- You may feel anxious or emotional distress while awaiting results. Some people would rather not know about their risk.
- It is also possible for your risk to be overestimated or underestimated. If your risk is overestimated, it may cause you anxiety and lead you have tests or treatment which may not have been necessary.
- When it comes to the way risk is predicted, some people find mammography uncomfortable and painful. If risk is predicted from a questionnaire then it depends on your answers and if inaccurate information is provided, this might lead to a inaccurate risk estimate.
- If a genetic test is used as part of your risk prediction the results may have implications for your family. For example, the test might suggest that your children or close relatives could also be at higher risk of developing breast cancer in their lifetime.

dceintro

We will now ask you some questions about your preferences for a potential service for predicting your risk of breast cancer.

In the next section you will be shown 10 questions. In each question we will show you two potential risk prediction services that could be offered. These services will be described using 6 characteristics:

- How your risk of breast cancer would be predicted
- How many appointments would be needed to predict your risk of breast cancer
- Where you would need to go to have your risk predicted
- What days and time of day appointments would be available
- How you would be able to book an appointment
- The likelihood that you would be predicted to be at high risk of breast cancer

In each question you will be asked which of the two services you would prefer if you had to choose or whether

you would choose not to have your risk of breast cancer predicted. We are interested in your views even if you would never want to have your risk of breast cancer predicted. The image below shows an example of how these risk prediction services would be presented:

	**Risk Prediction 1**	**Risk Prediction 2**	**No Risk Prediction**
**How Risk is Predicted**	Questionnaire and genetic test	Questionnaire and radiofrequency scan	
**How Many Appointments are Needed**	Two	One	You would not have your risk of breast cancer predicted
**Location of Appointment**	Mobile Van	Community Centre	You would be invited to breast cancer screening at age 50
**Possible Times for Appointment**	Weekdays, Evenings and Weekends	Weekdays only	If you were worried about cancer before this, you would visit your GP
**How Appointments are Booked**	Book a time yourself online or on the phone	Receive a letter with a fixed time	
**The likelihood you would be estimated to be at high risk**	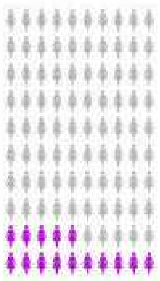 15 out of 100 would be predicted to be at higher risk of breast cancer; 85 out of 100 would be predicted to be at normal risk of breast cancer	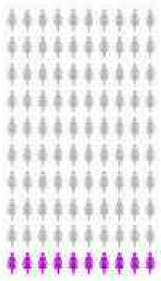 10 out of 100 would be predicted to be at higher risk of breast cancer; 90 out of 100 would be predicted to be at normal risk of breast cancer	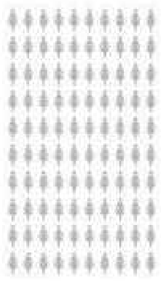 0 out of 100 would be predicted to be at higher risk of breast cancer; 100 out of 100 would be predicted to be at normal risk of breast cancer

In the next pages we'll give you some more information about each of the characteristics of the service and then show you an example question

How your risk of breast cancer could be provided

In the potential breast cancer risk prediction services we will show you, you're risk could be calculated using:

- A questionnaire only: This questionnaire would ask you details about yourself such as your age, height, weight, number of children and age of first period. It will also ask you about your family history
- A questionnaire and breast density mammography: This would involve completing the questionnaire and having a mammography to check the density of your breasts. This would be a low-dose mammography using a lower level of radiation. It would not find any cancers which may be present.
- A questionnaire and radiofrequency scan. This would involve completing the questionnaire and have a radiofrequency scan to check your breast density, Radiofrequency scanners are handheld devices which are portable and can check breast density without the use of radiation.
- A questionnaire, breast density mammography, and genetic test: This would involve completing the questionnaire, having a mammography and providing a sample of saliva (spit). This would be sent for genetic testing and the information added to your risk prediction.
- A questionnaire and genetic test: This would involve completing the questionnaire and providing a saliva sample for testing but would not involve having a mammography.

- A questionnaire, radiofrequency scan, and genetic test. This would involve completing the questionnaire, having a radiofrequency scan to check your breast density, and providing a saliva sample for genetic testing.

How many appointments would be needed to predict your risk

In some of the potential risk prediction services you would only need to attend one appointment to have your risk predicted. However, in others it may be necessary to attend two appointments on different days.

Where you would need to go to have your risk predicted

The risk prediction service could be offered in different places. This may depend on how your risk is predicted. The different potential locations are:

- Your own home
- Your general practitioner (GP)
- A mobile van at your local supermarket
- Your nearest hospital
- A local community centre

What days and time of day appointments would be available

Appointments to have your risk predicted could be made available at different times. Some services might be available from 9-5 on weekdays. In other services appointments may be available between 9-5 on weekdays but also in the evening or at the weekend.

How you can book an appointment

Different services could use different approaches to booking an appointment. In some services you would be sent a letter with a fixed date a time for your appointment. In other services you may be able to book a date and time of your choosing online or by phone.

The likelihood that your are predicted to be at high risk of breast cancer

Different ways of predicting risk might result in different numbers of women being predicted to be at higher risk and receiving early screening or preventative treatment.

The chance that you would be predicted to be at high risk

could vary in different risk prediction services. This chance could be:

- 5 out of every 100 people (5%) of women would be predicted to be at high risk
- 10 out of every 100 people (10%) of women would be predicted to be at high risk
- 15 out of every 100 people (15%) of women would be predicted to be at high risk
- 20 out of every 100 people (20%) of women would be predicted to be at high risk

We'll now show you a series of questions in which we will ask you to choose which risk prediction service you would prefer. Please remember we are interested in hearing your views even if you don't think you would like to have your risk of breast cancer predicted.

BCAN block 1 v2

If you had to choose between the following breast cancer risk prediction services, which would you choose? You can also choose not to receive breast cancer risk prediction

	Risk Prediction 1	Risk Prediction 2	No Risk Prediction
**How Risk is Predicted**	Questionnaire	Questionnaire and genetic test	
**How Many Appointments are Needed**	One	One	You would not have your risk of breast cancer predicted
**Location of Appointment**	Home	Home	You would be invited to breast cancer screening at age 50
**Possible Times for Appointment**	Weekdays, Evenings and Weekends	Weekdays, Evenings and Weekends	If you were worried about cancer

	Risk Prediction 1	Risk Prediction 2	No Risk Prediction
			before this, you would visit your GP
**How Appointments are Booked**	Receive a letter with a fixed time	Book a time yourself online or on the phone	
**The likelihood you would be estimated to be at high risk**	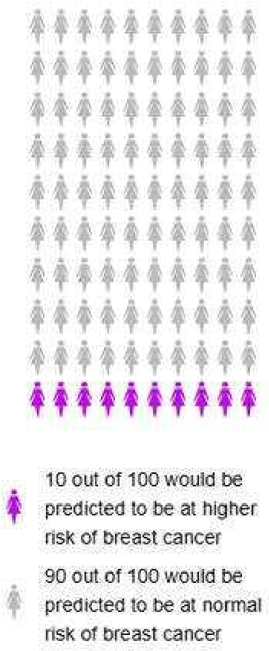	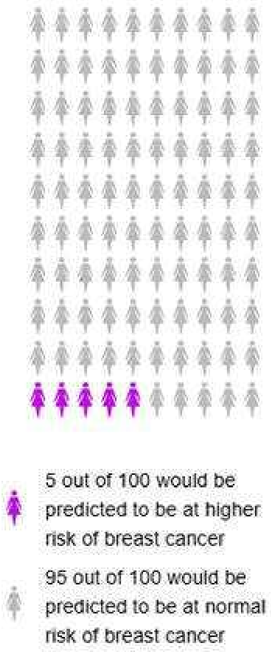	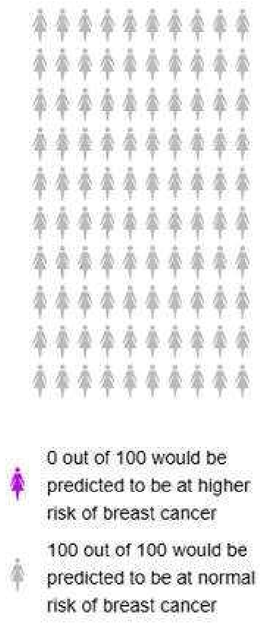

	Test 1	Test 2	No Risk Prediction
Your choice:	○	○	○

If you had to choose between the following breast cancer risk prediction services, which would you choose? You can also choose not to receive breast cancer risk prediction

	Risk Prediction 1	Risk Prediction 2	No Risk Prediction
**How Risk is Predicted**	Questionnaire, mammography scan and genetic test	Questionnaire and mammography scan	
**How Many Appointments are Needed**	One	Two	You would not have your risk of breast cancer predicted
**Location of Appointment**	Hospital	Hospital	You would be invited tc breast cancer screening at age 50
**Possible Times for Appointment**	Weekdays only	Weekdays only	If you were worried about cancer

	**Risk Prediction 1**	**Risk Prediction 2**	**No Risk Prediction**
			before this, you would visit your GP
**How Appointments are Booked**	Book a time yourself online or on the phone	Receive a letter with a fixed time	
**The likelihood you would be estimated to be at high risk**	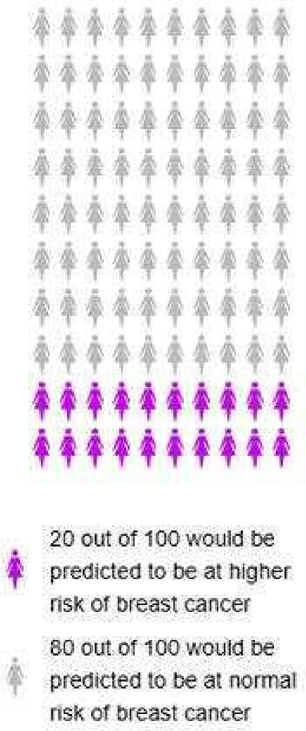	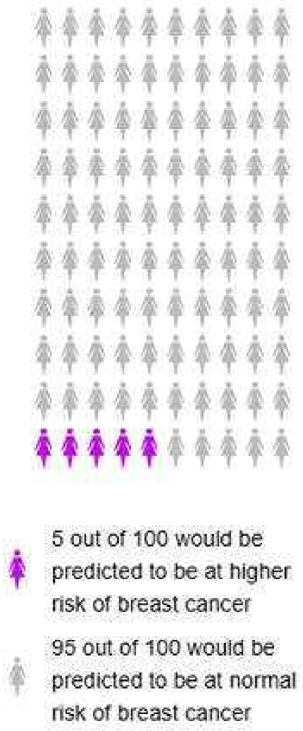	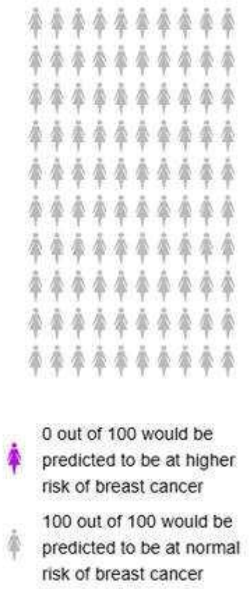

	Test 1	Test 2	No Risk Prediction
Your choice:	○	○	○

If you had to choose between the following breast cancer risk prediction services, which would you choose? You

can also choose not to receive breast cancer risk prediction

	Risk Prediction 1	Risk Prediction 2	No Risk Prediction
**How Risk is Predicted**	Questionnaire, radiofrequency scan, and genetic test	Questionnaire and genetic test	
**How Many Appointments are Needed**	Two	Two	You would not have your risk of breast cancer predicted
**Location of Appointment**	Your GP	Your GP	You would be invited to breast cancer screening at age 50
**Possible Times for Appointment**	Weekdays only	Weekdays, Evenings and Weekends	If you were worried about cancer before this,

	**Risk Prediction 1**	**Risk Prediction 2**	**No Risk Prediction**
			you would visit your GP
**How Appointments are Booked**	Receive a letter with a fixed time	Receive a letter with a fixed time	
**The likelihood you would be estimated to be at high risk**	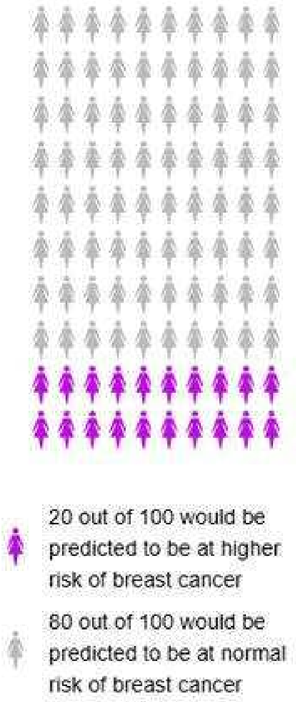	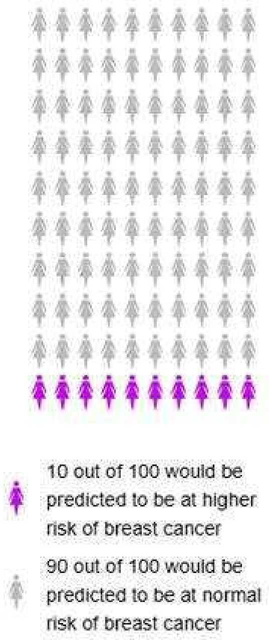	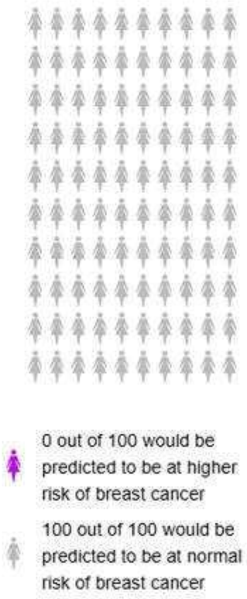

	Test 1	Test 2	No Risk Prediction
Your choice:	○	○	○

If you had to choose between the following breast cancer risk prediction services, which would you choose? You

can also choose not to receive breast cancer risk prediction

	Risk Prediction 1	Risk Prediction 2	No Risk Prediction
**How Risk is Predicted**	Questionnaire and radiofrequency scan	Questionnaire and mammography scan	
**How Many Appointments are Needed**	One	Two	You would not have your risk of breast cancer predicted
**Location of Appointment**	Mobile Van	Mobile Van	You would be invited to breast cancer screening at age 50
**Possible Times for Appointment**	Weekdays, Evenings and Weekends	Weekdays only	If you were worried about cancer before this, you would visit your GP

	**Risk Prediction 1**	**Risk Prediction 2**	**No Risk Prediction**
**How Appointments are Booked**	Receive a letter with a fixed time	Book a time yourself online or on the phone	
**The likelihood you would be estimated to be at high risk**	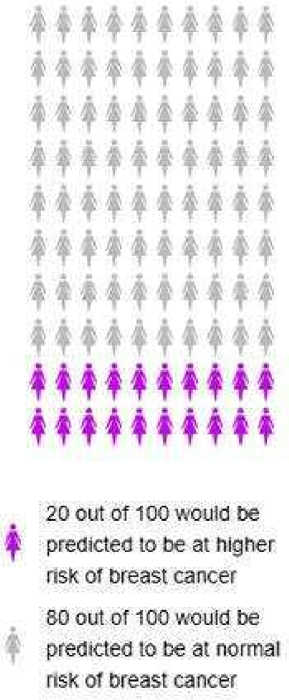	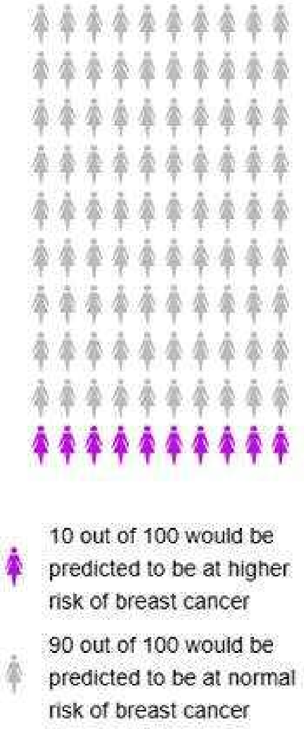	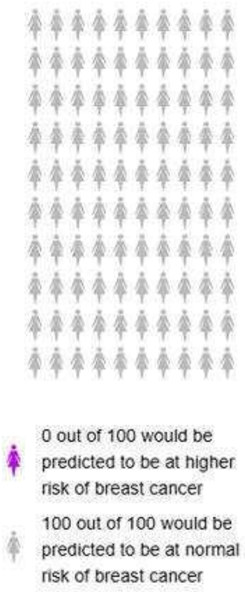

	Test 1	Test 2	No Risk Prediction
Your choice:	○	○	○

If you had to choose between the following breast cancer risk prediction services, which would you choose? You can also choose not to receive breast cancer risk prediction

	Risk Prediction 1	Risk Prediction 2	No Risk Prediction
**How Risk is Predicted**	Questionnaire and mammography scan	Questionnaire, radiofrequency scan, and genetic test	
**How Many Appointments are Needed**	Two	Two	You would not have your risk of breast cancer predicted
**Location of Appointment**	Hospital	Mobile Van	You would be invited to breast cancer screening at age 50
**Possible Times for Appointment**	Weekdays, Evenings and Weekends	Weekdays, Evenings and Weekends	If you were worried about cancer before this, you would visit your GP
**How Appointments**	Book a time yourself online	Receive a letter with a	

	**Risk Prediction 1**	**Risk Prediction 2**	**No Risk Prediction**
**are Booked**	or on the phone	fixed time	
**The likelihood you would be estimated to be at high risk**	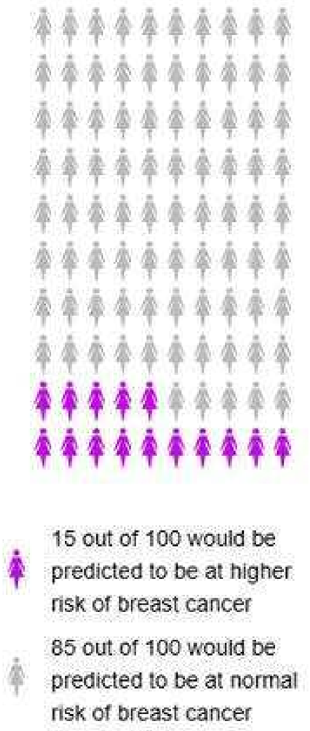	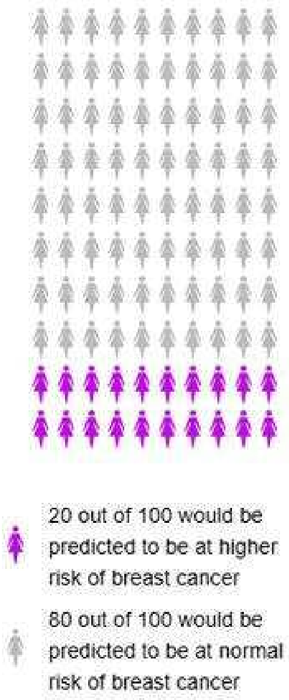	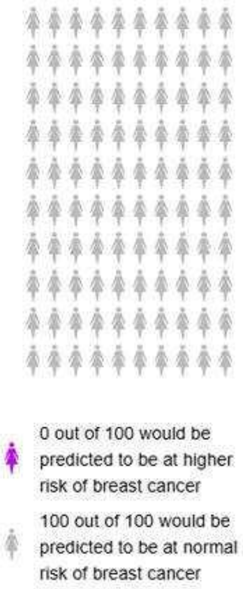

	Test 1	Test 2	No Risk Prediction
Your choice:	○	○	○

If you had to choose between the following breast cancer risk prediction services, which would you choose? You can also choose not to receive breast cancer risk prediction

	Risk Prediction 1	Risk Prediction 2	No Risk Prediction
**How Risk is Predicted**	Questionnaire	Questionnaire	You would not have your risk of breast cancer predicted
**How Many Appointments are Needed**	One	One	
**Location of Appointment**	Home	Your GP	You would be invited to breast cancer screening at age 50
**Possible Times for Appointment**	Weekdays, Evenings and Weekends	Weekdays, Evenings and Weekends	If you were worried about cancer before this, you would visit your GP
**How Appointments are Booked**	Book a time yourself	Book a time yourself	

	**Risk Prediction 1**	**Risk Prediction 2**	**No Risk Prediction**
	online or on the phone	online or on the phone	
**The likelihood you would be estimated to be at high risk**	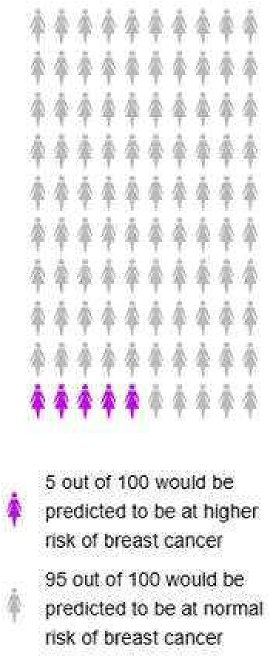	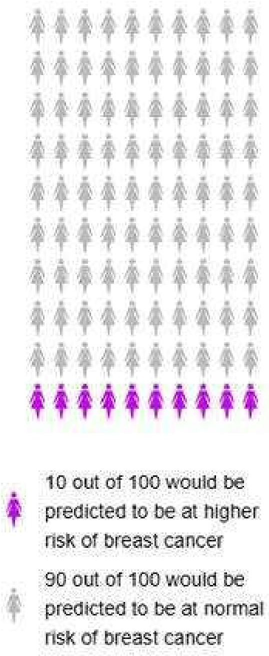	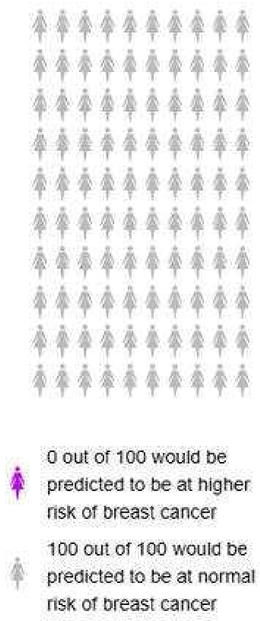

	Test 1	Test 2	No Risk Prediction
Your choice:	○	○	○

If you had to choose between the following breast cancer risk prediction services, which would you choose? You can also choose not to receive breast cancer risk prediction

	Risk Prediction 1	Risk Prediction 2	No Risk Prediction
**How Risk is Predicted**	Questionnaire and genetic test	Questionnaire and radiofrequency scan	
**How Many Appointments are Needed**	Two	One	You would not have your risk of breast cancer predicted
**Location of Appointment**	Mobile Van	Community Centre	You would be invited to breast cancer screening at age 50
**Possible Times for Appointment**	Weekdays, Evenings and Weekends	Weekdays only	If you were worried about cancer before this, you would visit your GP

	**Risk Prediction 1**	**Risk Prediction 2**	**No Risk Prediction**
**How Appointments are Booked**	Book a time yourself online or on the phone	Receive a letter with a fixed time	
**The likelihood you would be estimated to be at high risk**	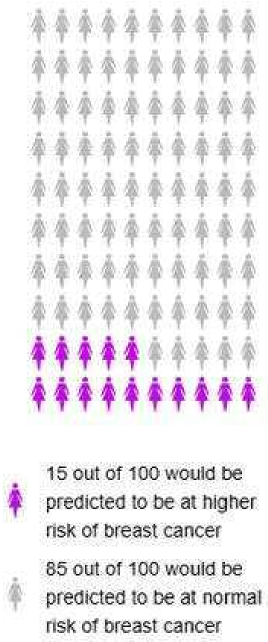	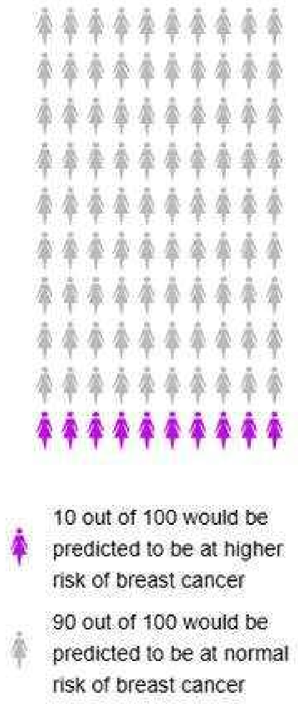	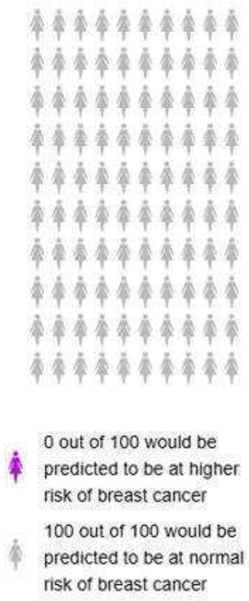

	Test 1	Test 2	No Risk Prediction
Your choice:	○	○	○

If you had to choose between the following breast cancer risk prediction services, which would you choose? You

can also choose not to receive breast cancer risk prediction

	Risk Prediction 1	Risk Prediction 2	No Risk Prediction
**How Risk is Predicted**	Questionnaire	Questionnaire, mammography scan and genetic test	
**How Many Appointments are Needed**	One	One	You would not have your risk of breast cancer predicted
**Location of Appointment**	Your GP	Mobile Van	You would be invited to breast cancer screening at age 50
**Possible Times for Appointment**	Weekdays only	Weekdays only	If you were worried about cancer before this,

	**Risk Prediction 1**	**Risk Prediction 2**	**No Risk Prediction**
			you would visit your GP
**How Appointments are Booked**	Receive a letter with a fixed time	Book a time yourself online or on the phone	
**The likelihood you would be estimated to be at high risk**	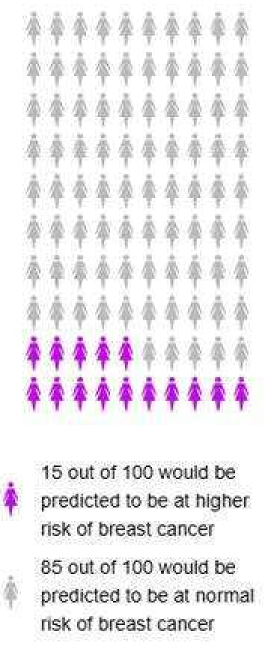	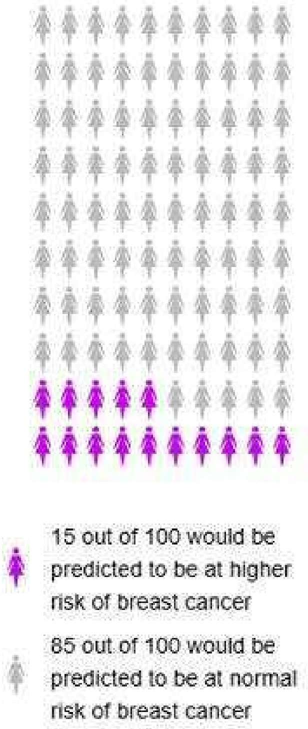	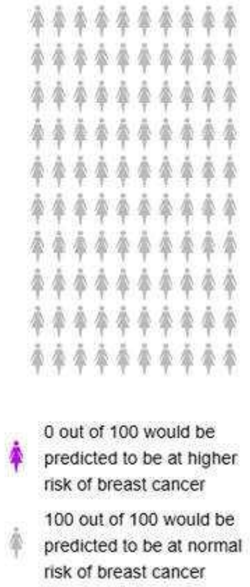

	Test 1	Test 2	No Risk Prediction
Your choice:	○	○	○

If you had to choose between the following breast cancer risk prediction services, which would you choose? You

can also choose not to receive breast cancer risk prediction

	Risk Prediction 1	Risk Prediction 2	No Risk Prediction
**How Risk is Predicted**	Questionnaire and radiofrequency scan	Questionnaire, radiofrequency scan, and genetic test	
**How Many Appointments are Needed**	One	Two	You would not have your risk of breast cancer predicted
**Location of Appointment**	Community Centre	Community Centre	You would be invited to breast cancer screening at age 50
**Possible Times for Appointment**	Weekdays, Evenings and Weekends	Weekdays, Evenings and Weekends	If you were worried about cancer before this, you would visit your GP

	Risk Prediction 1	Risk Prediction 2	No Risk Prediction
**How Appointments are Booked**	Book a time yourself online or on the phone	Book a time yourself online or on the phone	
**The likelihood you would be estimated to be at high risk**	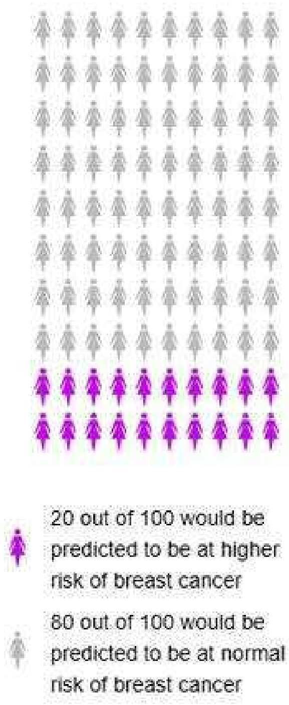	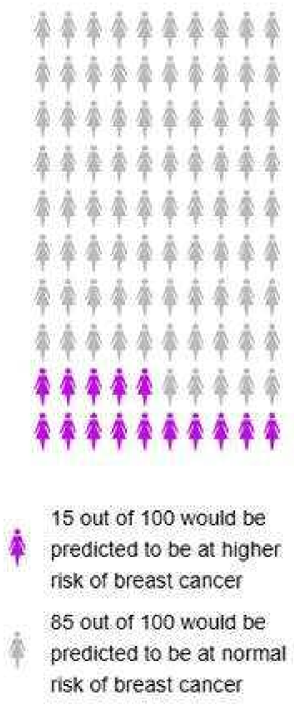	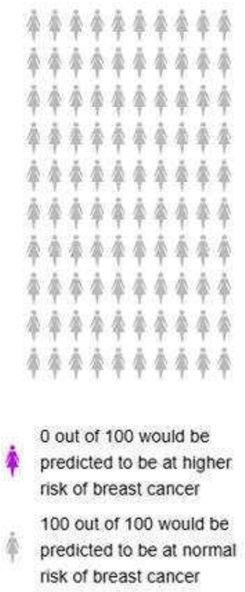

	Test 1	Test 2	No Risk Prediction
Your choice:	○	○	○

If you had to choose between the following breast cancer risk prediction services, which would you choose? You can also choose not to receive breast cancer risk prediction

	Risk Prediction 1	Risk Prediction 2	No Risk Prediction
**How Risk is Predicted**	Questionnaire and genetic test	Questionnaire and genetic test	
**How Many Appointments are Needed**	One	One	You would not have your risk of breast cancer predicted
**Location of Appointment**	Hospital	Hospital	You would be invited to breast cancer screening at age 50
**Possible Times for Appointment**	Weekdays, Evenings and Weekends	Weekdays only	If you were worried about cancer before this, you would visit your GP
**How Appointments**	Receive a letter with a	Book a time yourself	

	**Risk Prediction 1**	**Risk Prediction 2**	**No Risk Prediction**
**are Booked**	fixed time	online or on the phone	
**The likelihood you would be estimated to be at high risk**	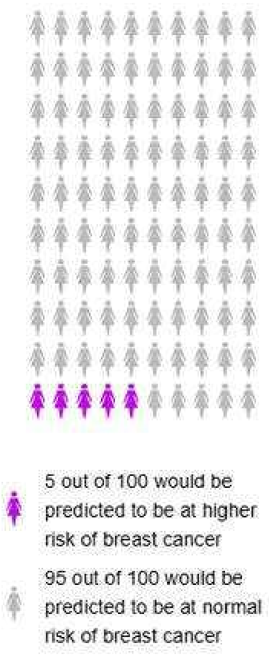	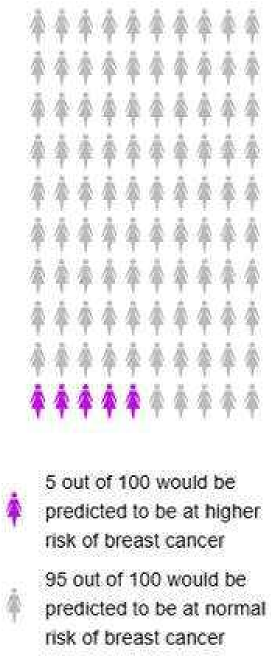	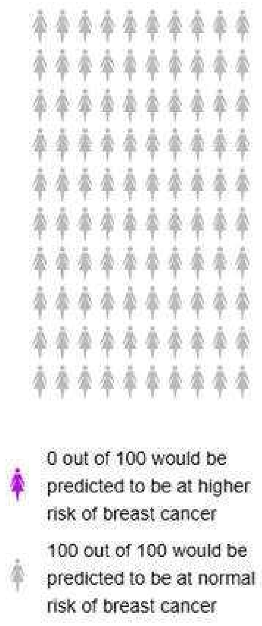

	Test 1	Test 2	No Risk Prediction
Your choice:	○	○	○

BCAN block 2 v2

If you had to choose between the following breast cancer risk prediction services, which would you choose? You can also choose not to receive breast cancer risk prediction

	Risk Prediction 1	Risk Prediction 2	No Risk Prediction
**How Risk is Predicted**	Questionnaire, radiofrequency scan, and genetic test	Questionnaire, mammography scan and genetic test	
**How Many Appointments are Needed**	One	Two	You would not have your risk of breast cancer predicted
**Location of Appointment**	Hospital	Hospital	You would be invited to breast cancer screening at age 50
**Possible Times for Appointment**	Weekdays only	Weekdays, Evenings and Weekends	If you were worried about cancer before this, you would visit your GP
**How Appointments**	Book a time yourself online	Receive a letter with a fixed	

	Risk Prediction 1	Risk Prediction 2	No Risk Prediction
**are Booked**	or on the phone	time	
**The likelihood you would be estimated to be at high risk**	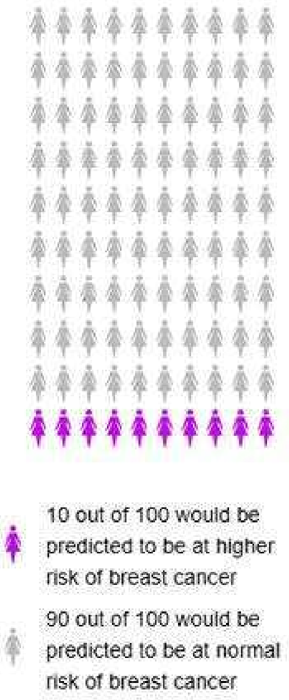	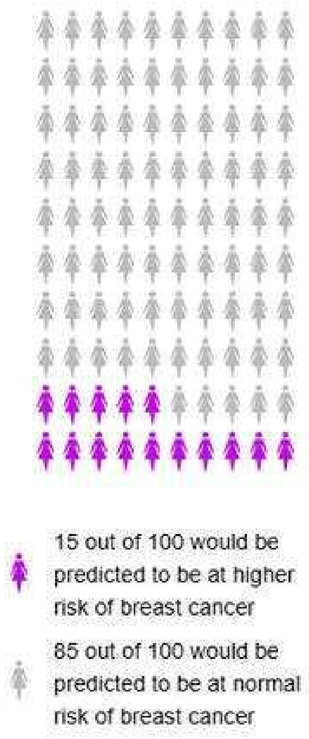	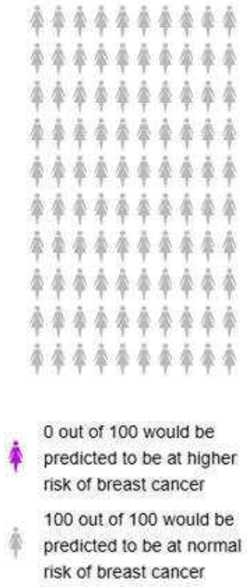

	Test 1	Test 2	No Risk Prediction
Your choice:	○	○	○

If you had to choose between the following breast cancer risk prediction services, which would you choose? You can also choose not to receive breast cancer risk prediction

	Risk Prediction 1	Risk Prediction 2	No Risk Prediction
**How Risk is Predicted**	Questionnaire and radiofrequency scan	Questionnaire and genetic test	
**How Many Appointments are Needed**	One	Two	You would not have your risk of breast cancer predicted
**Location of Appointment**	Your GP	Community Centre	You would be invited to breast cancer screening at age 50
**Possible Times for Appointment**	Weekdays only	Weekdays, Evenings and Weekends	If you were worried about cancer before this, you would visit your GP

<table>
<tr><th></th><th>Risk Prediction 1</th><th>Risk Prediction 2</th><th>No Risk Prediction</th></tr>
<tr><td>How Appointments are Booked</td><td>Receive a letter with a fixed time</td><td>Book a time yourself online or on the phone</td><td></td></tr>
<tr><td>The likelihood you would be estimated to be at high risk</td><td>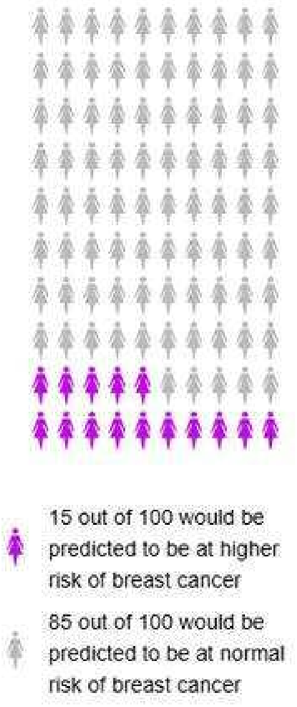</td><td>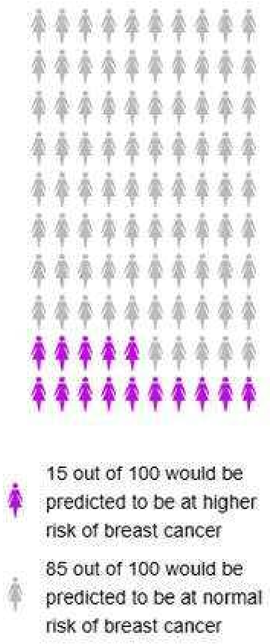</td><td>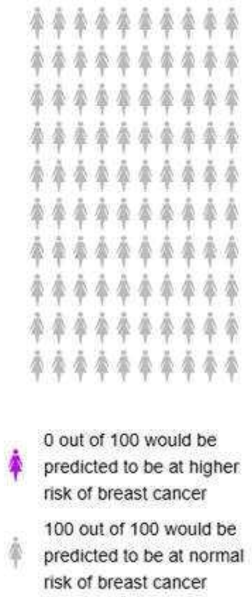</td></tr>
</table>

	Test 1	Test 2	No Risk Prediction
Your choice:	○	○	○

If you had to choose between the following breast cancer risk prediction services, which would you choose? You

can also choose not to receive breast cancer risk prediction

	Risk Prediction 1	Risk Prediction 2	No Risk Prediction
**How Risk is Predicted**	Questionnaire and radiofrequency scan	Questionnaire	
**How Many Appointments are Needed**	Two	One	You would not have your risk of breast cancer predicted
**Location of Appointment**	Hospital	Mobile Van	You would be invited to breast cancer screening at age 50
**Possible Times for Appointment**	Weekdays only	Weekdays only	If you were worried about cancer before this,

	**Risk Prediction 1**	**Risk Prediction 2**	**No Risk Prediction**
**How Appointments are Booked**	Book a time yourself online or on the phone	Receive a letter with a fixed time	you would visit your GP
**The likelihood you would be estimated to be at high risk**	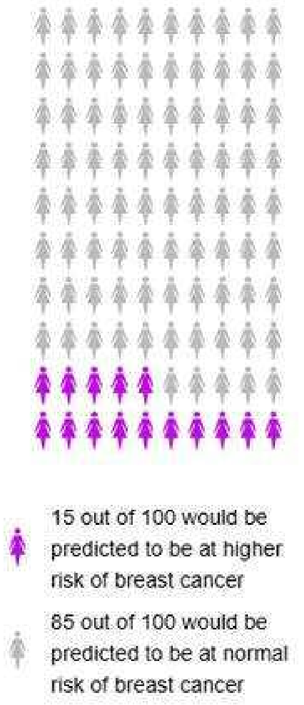	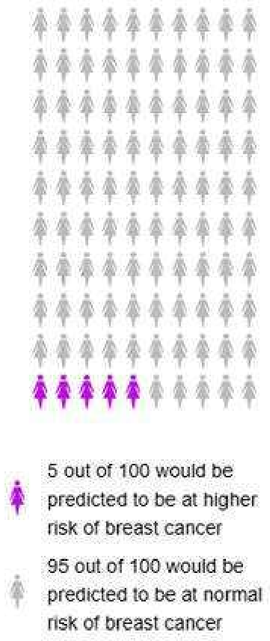	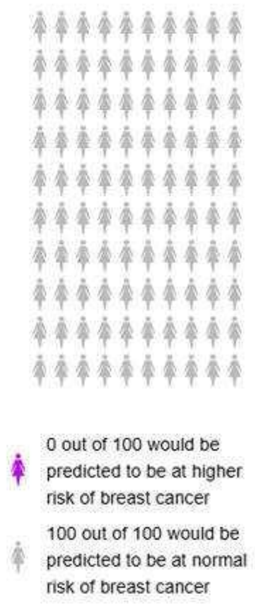

	Test 1	Test 2	No Risk Prediction
Your choice:	○	○	○

If you had to choose between the following breast cancer risk prediction services, which would you choose? You can also choose not to receive breast cancer risk prediction

	Risk Prediction 1	Risk Prediction 2	No Risk Prediction
**How Risk is Predicted**	Questionnaire and genetic test	Questionnaire and mammography scan	
**How Many Appointments are Needed**	One	One	You would not have your risk of breast cancer predicted
**Location of Appointment**	Your GP	Mobile Van	You would be invited to breast cancer screening at age 50
**Possible Times for Appointment**	Weekdays, Evenings and Weekends	Weekdays, Evenings and Weekends	If you were worried about

	Risk Prediction 1	Risk Prediction 2	No Risk Prediction
			cancer before this, you would visit your GP
**How Appointments are Booked**	Receive a letter with a fixed time	Book a time yourself online or on the phone	
**The likelihood you would be estimated to be at high risk**	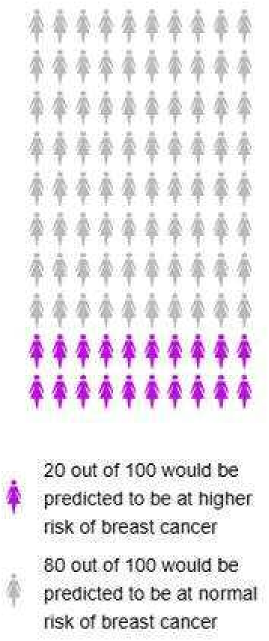	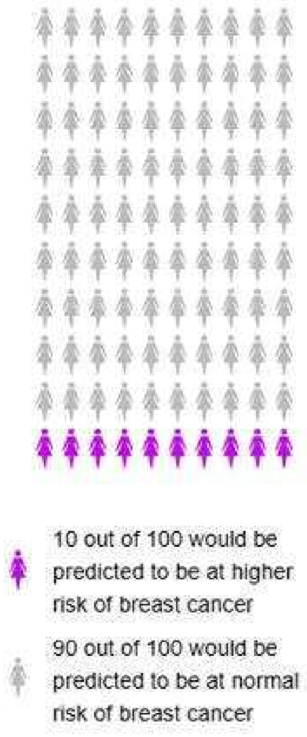	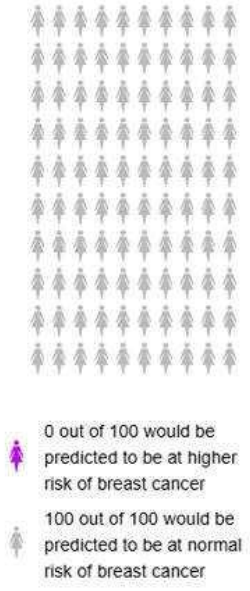

	Test 1	Test 2	No Risk Prediction
Your choice:	○	○	○

If you had to choose between the following breast cancer risk prediction services, which would you choose? You can also choose not to receive breast cancer risk prediction

	**Risk Prediction 1**	**Risk Prediction 2**	**No Risk Prediction**
**How Risk is Predicted**	Questionnaire, radiofrequency scan, and genetic test	Questionnaire, mammography scan and genetic test	
**How Many Appointments are Needed**	One	Two	You would not have your risk of breast cancer predicted
**Location of Appointment**	Community Centre	Mobile Van	You would be invited to breast cancer screening at age 50
**Possible Times for Appointment**	Weekdays, Evenings and Weekends	Weekdays only	If you were worried about cancer

	Risk Prediction 1	Risk Prediction 2	No Risk Prediction
**How Appointments are Booked**	Book a time yourself online or on the phone	Book a time yourself online or on the phone	before this, you would visit your GP
**The likelihood you would be estimated to be at high risk**	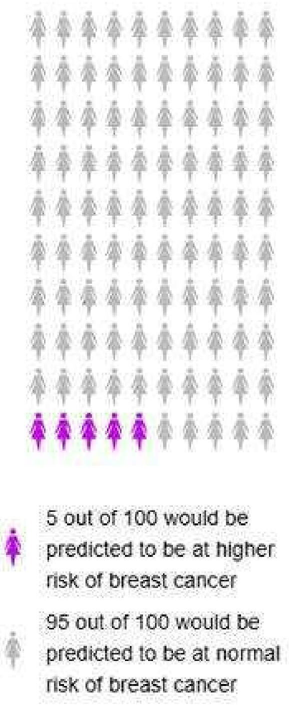	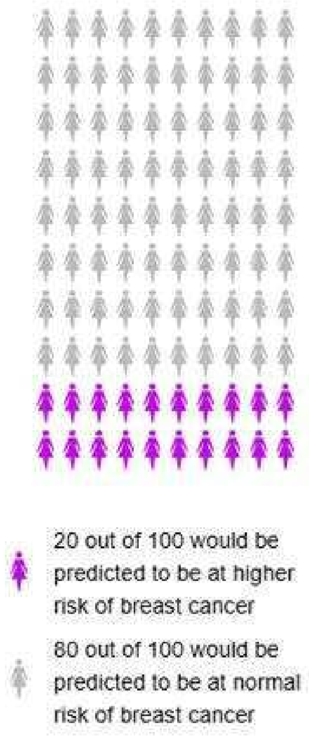	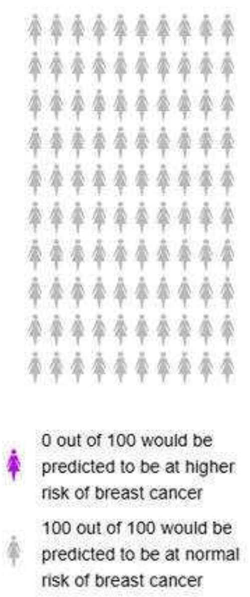

	Test 1	Test 2	No Risk Prediction
Your choice:	○	○	○

If you had to choose between the following breast cancer risk prediction services, which would you choose? You can also choose not to receive breast cancer risk prediction

	**Risk Prediction 1**	**Risk Prediction 2**	**No Risk Prediction**
**How Risk is Predicted**	Questionnaire and radiofrequency scan	Questionnaire, radiofrequency scan, and genetic test	
**How Many Appointments are Needed**	Two	Two	You would not have your risk of breast cancer predicted
**Location of Appointment**	Community Centre	Community Centre	You would be invited to breast cancer screening at age 50
**Possible Times for Appointment**	Weekdays only	Weekdays, Evenings and Weekends	If you were worried about cancer

	**Risk Prediction 1**	**Risk Prediction 2**	**No Risk Prediction**
			before this, you would visit your GP
**How Appointments are Booked**	Receive a letter with a fixed time	Receive a letter with a fixed time	
**The likelihood you would be estimated to be at high risk**	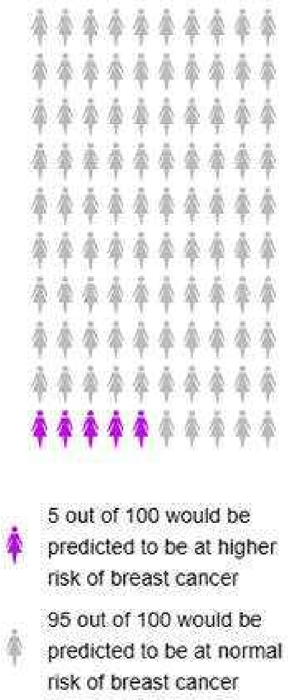	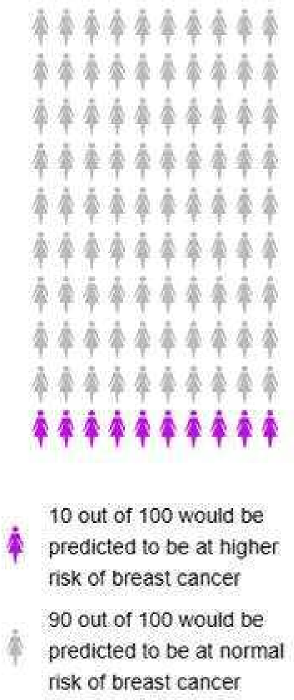	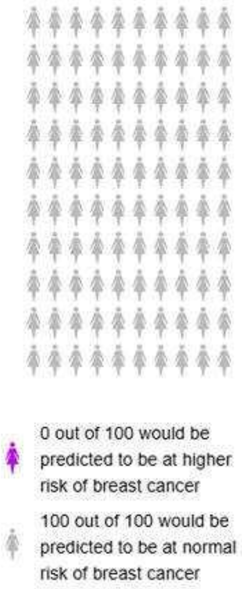

	Test 1	Test 2	No Risk Prediction
Your choice:	○	○	○

If you had to choose between the following breast cancer risk prediction services, which would you choose? You

can also choose not to receive breast cancer risk prediction

	Risk Prediction 1	Risk Prediction 2	No Risk Prediction
**How Risk is Predicted**	Questionnaire, mammography scan and genetic test	Questionnaire and radiofrequency scan	
**How Many Appointments are Needed**	Two	Two	You would not have your risk of breast cancer predicted
**Location of Appointment**	Hospital	Your GP	You would be invited to breast cancer screening at age 50
**Possible Times for Appointment**	Weekdays, Evenings and Weekends	Weekdays, Evenings and Weekends	If you were worried about cancer before this, you would visit your GP

	**Risk Prediction 1**	**Risk Prediction 2**	**No Risk Prediction**
**How Appointments are Booked**	Receive a letter with a fixed time	Book a time yourself online or on the phone	

The likelihood you would be estimated to be at high risk

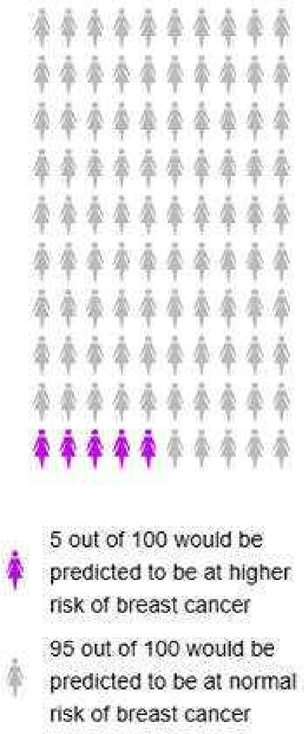

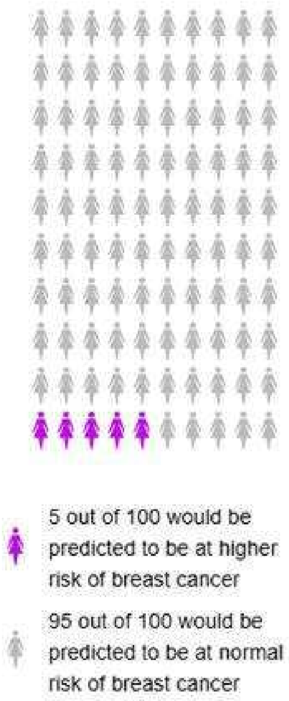

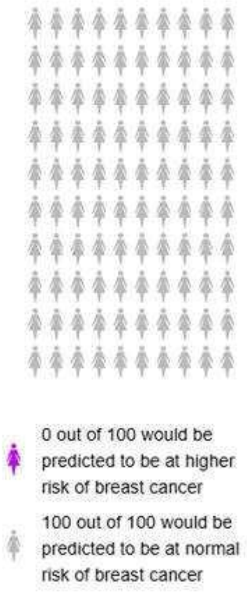

	Test 1	Test 2	No Risk Prediction
Your choice:	○	○	○

If you had to choose between the following breast cancer risk prediction services, which would you choose? You can also choose not to receive breast cancer risk prediction

	Risk Prediction 1	Risk Prediction 2	No Risk Prediction
**How Risk is Predicted**	Questionnaire and genetic test	Questionnaire and genetic test	
**How Many Appointments are Needed**	Two	One	You would not have your risk of breast cancer predicted
**Location of Appointment**	Your GP	Home	You would be invited to breast cancer screening at age 50
**Possible Times for Appointment**	Weekdays only	Weekdays, Evenings and Weekends	If you were worried about cancer before this, you would visit your GP
**How Appointments**	Book a time yourself	Receive a letter with a	

	**Risk Prediction 1**	**Risk Prediction 2**	**No Risk Prediction**
**are Booked**	online or on the phone	fixed time	
**The likelihood you would be estimated to be at high risk**	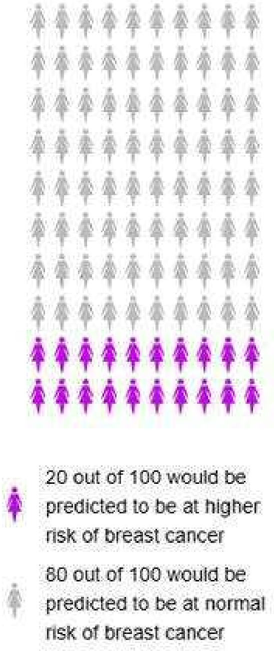	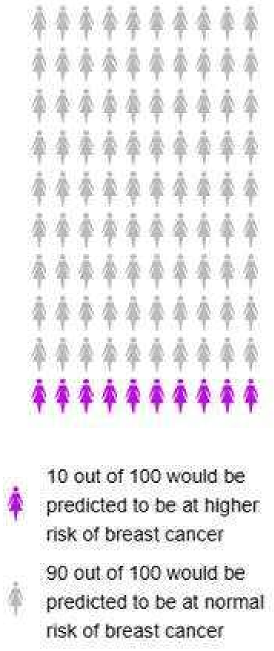	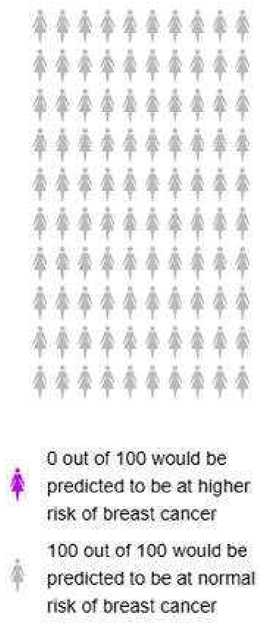

	Test 1	Test 2	No Risk Prediction
Your choice:	○	○	○

If you had to choose between the following breast cancer risk prediction services, which would you choose? You can also choose not to receive breast cancer risk prediction

	Risk Prediction 1	Risk Prediction 2	No Risk Prediction
**How Risk is Predicted**	Questionnaire	Questionnaire, radiofrequency scan, and genetic test	
**How Many Appointments are Needed**	One	One	You would not have your risk of breast cancer predicted
**Location of Appointment**	Mobile Van	Hospital	You would be invited to breast cancer screening at age 50
**Possible Times for Appointment**	Weekdays, Evenings and Weekends	Weekdays only	If you were worried about cancer before this, you would visit your GP

	Risk Prediction 1	Risk Prediction 2	No Risk Prediction
**How Appointments are Booked**	Book a time yourself online or on the phone	Receive a letter with a fixed time	
**The likelihood you would be estimated to be at high risk**	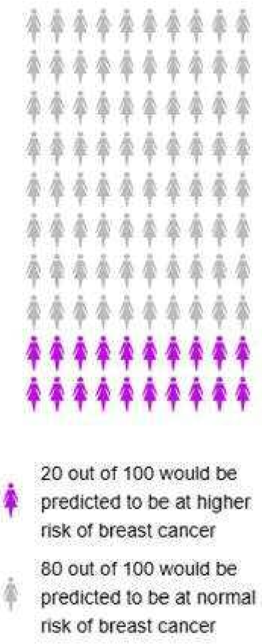	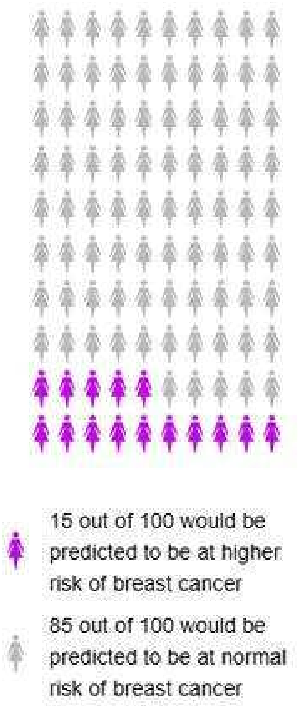	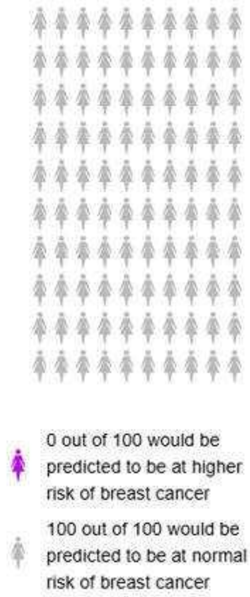

	Test 1	Test 2	No Risk Prediction
Your choice:	○	○	○

If you had to choose between the following breast cancer risk prediction services, which would you choose? You

can also choose not to receive breast cancer risk prediction

	**Risk Prediction 1**	**Risk Prediction 2**	**No Risk Prediction**
**How Risk is Predicted**	Questionnaire	Questionnaire and mammography scan	
**How Many Appointments are Needed**	One	One	You would not have your risk of breast cancer predicted
**Location of Appointment**	Home	Mobile Van	You would be invited to breast cancer screening at age 50
**Possible Times for Appointment**	Weekdays, Evenings and Weekends	Weekdays only	If you were worried about cancer before this,

	**Risk Prediction 1**	**Risk Prediction 2**	**No Risk Prediction**
			you would visit your GP
**How Appointments are Booked**	Book a time yourself online or on the phone	Receive a letter with a fixed time	
**The likelihood you would be estimated to be at high risk**	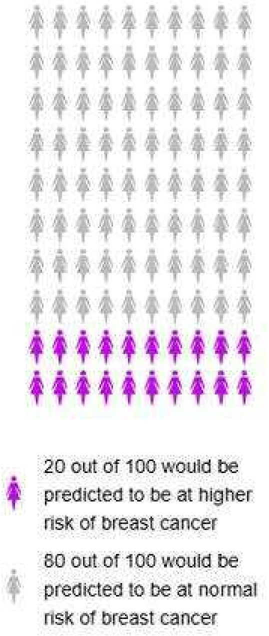	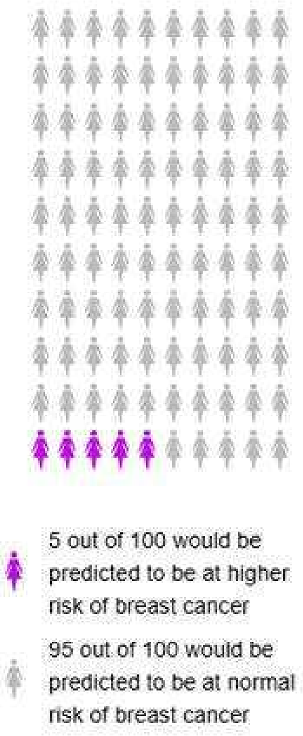	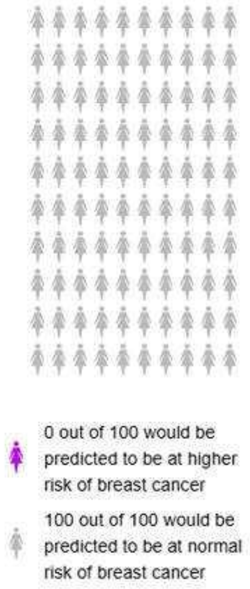

	Test 1	Test 2	No Risk Prediction
Your choice:	○	○	○

BCAN block 3 v2

If you had to choose between the following breast cancer risk prediction services, which would you choose? You can also choose not to receive breast cancer risk prediction

	Risk Prediction 1	Risk Prediction 2	No Risk Prediction
**How Risk is Predicted**	Questionnaire and radiofrequency scan	Questionnaire and genetic test	
**How Many Appointments are Needed**	One	Two	You would not have your risk of breast cancer predicted
**Location of Appointment**	Your GP	Home	You would be invited to breast cancer screening at age 50

<table>
<tr><th></th><th>Risk Prediction 1</th><th>Risk Prediction 2</th><th>No Risk Prediction</th></tr>
<tr><td>Possible Times for Appointment</td><td>Weekdays, Evenings and Weekends</td><td>Weekdays, Evenings and Weekends</td><td>If you were worried about cancer before this, you would visit your GP</td></tr>
<tr><td>How Appointments are Booked</td><td>Book a time yourself online or on the phone</td><td>Book a time yourself online or on the phone</td><td></td></tr>
<tr><td>The likelihood you would be estimated to be at high risk</td><td>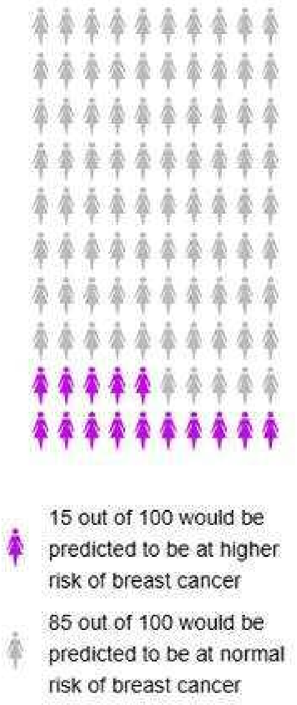
</td><td>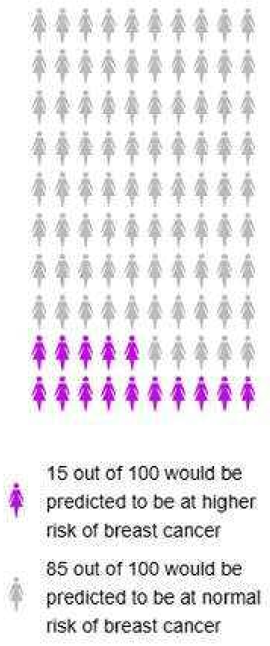
</td><td>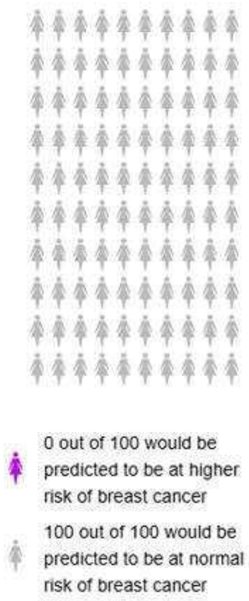
</td></tr>
</table>

	Test 1	Test 2	No Risk Prediction
Your choice:	○	○	○

If you had to choose between the following breast cancer risk prediction services, which would you choose? You can also choose not to receive breast cancer risk prediction

	**Risk Prediction 1**	**Risk Prediction 2**	**No Risk Prediction**
**How Risk is Predicted**	Questionnaire and mammography scan	Questionnaire and mammography scan	
**How Many Appointments are Needed**	Two	One	You would not have your risk of breast cancer predicted
**Location of Appointment**	Hospital	Mobile Van	You would be invited tc breast cancer screening at age 50

	**Risk Prediction 1**	**Risk Prediction 2**	**No Risk Prediction**
**Possible Times for Appointment**	Weekdays only	Weekdays, Evenings and Weekends	If you were worried about cancer before this, you would visit your GP
**How Appointments are Booked**	Receive a letter with a fixed time	Receive a letter with a fixed time	
**The likelihood you would be estimated to be at high risk**	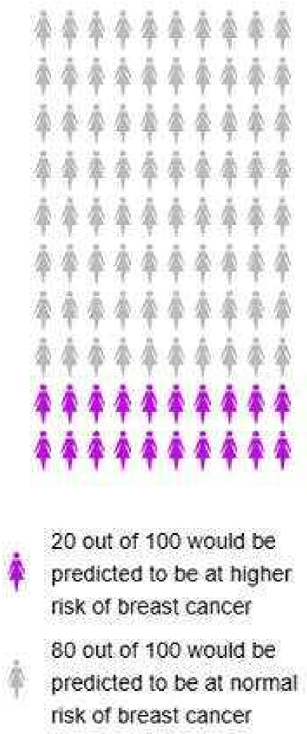	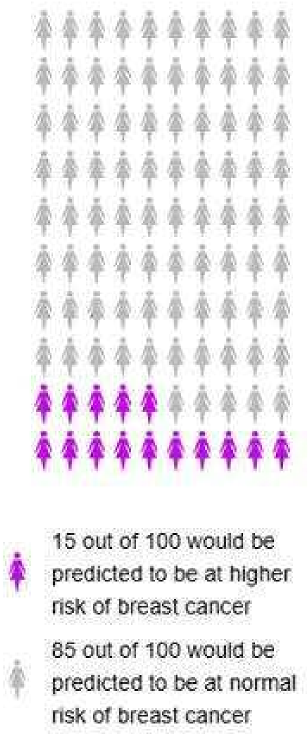	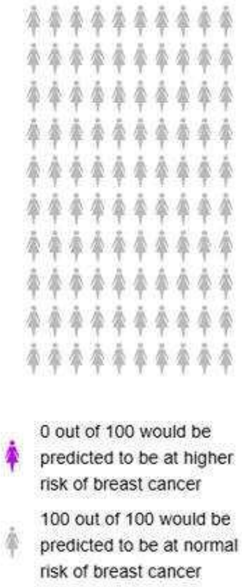

	Test 1	Test 2	No Risk Prediction
Your choice:	○	○	○

If you had to choose between the following breast cancer risk prediction services, which would you choose? You can also choose not to receive breast cancer risk prediction

	**Risk Prediction 1**	**Risk Prediction 2**	**No Risk Prediction**
**How Risk is Predicted**	Questionnaire	Questionnaire and genetic test	
**How Many Appointments are Needed**	One	Two	You would not have your risk of breast cancer predicted
**Location of Appointment**	Hospital	Home	You would be invited to breast cancer screening at age 50
**Possible Times for**	Weekdays, Evenings and	Weekdays, Evenings and	If you were worried

	Risk Prediction 1	Risk Prediction 2	No Risk Prediction
**Appointment**	Weekends	Weekends	about cancer before this, you would visit your GP
**How Appointments are Booked**	Receive a letter with a fixed time	Receive a letter with a fixed time	
**The likelihood you would be estimated to be at high risk**	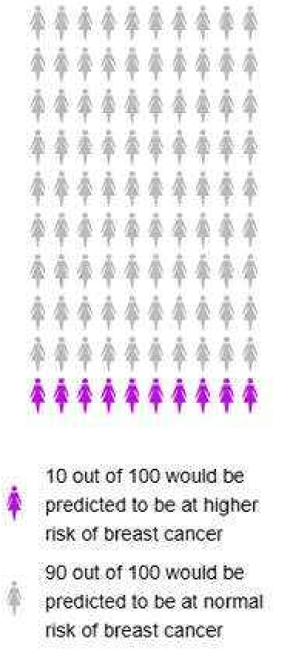	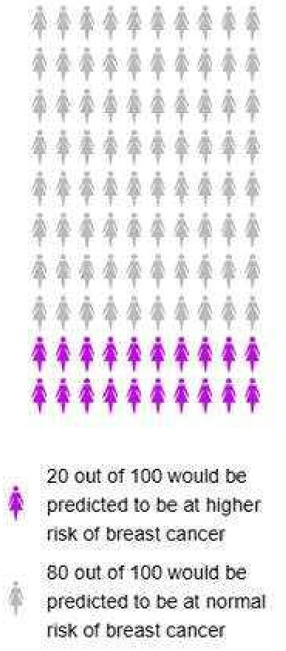	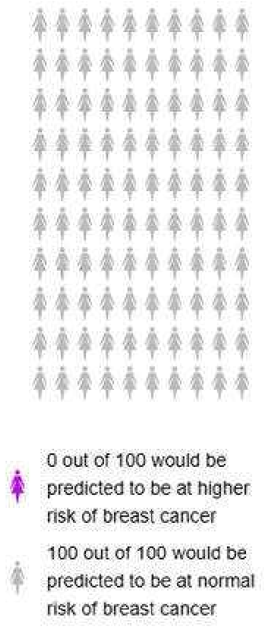

	Test 1	Test 2	No Risk Prediction
Your choice:	○	○	○

If you had to choose between the following breast cancer risk prediction services, which would you choose? You can also choose not to receive breast cancer risk prediction

	Risk Prediction 1	Risk Prediction 2	No Risk Prediction
**How Risk is Predicted**	Questionnaire and genetic test	Questionnaire	
**How Many Appointments are Needed**	Two	One	You would not have your risk of breast cancer predicted
**Location of Appointment**	Mobile Van	Community Centre	You would be invited to breast cancer screening at age 50
**Possible Times for Appointment**	Weekdays only	Weekdays only	If you were worried about cancer

	**Risk Prediction 1**	**Risk Prediction 2**	**No Risk Prediction**
			before this, you would visit your GP
**How Appointments are Booked**	Book a time yourself online or on the phone	Book a time yourself online or on the phone	
**The likelihood you would be estimated to be at high risk**	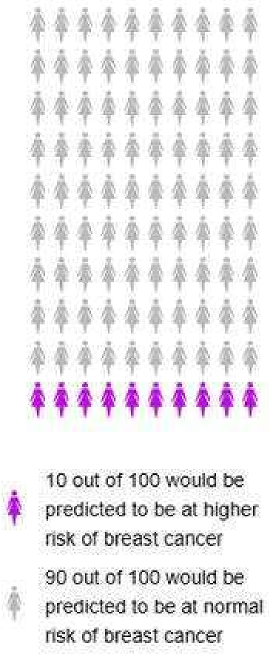	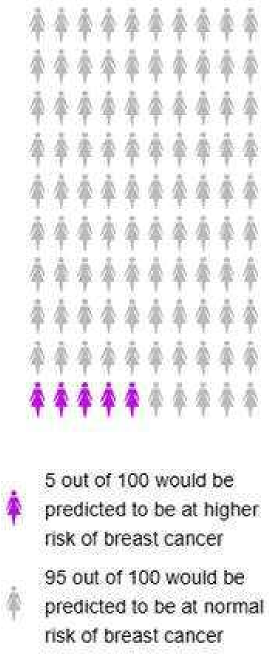	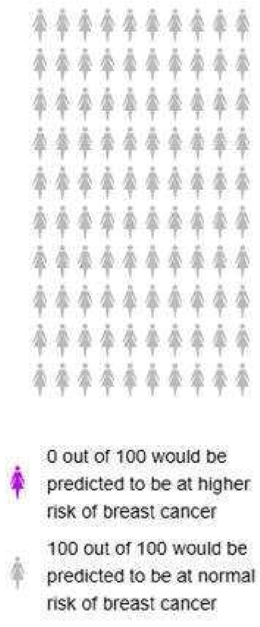

	Test 1	Test 2	No Risk Prediction
Your choice:	○	○	○

If you had to choose between the following breast cancer risk prediction services, which would you choose? You can also choose not to receive breast cancer risk prediction

	Risk Prediction 1	Risk Prediction 2	No Risk Prediction
**How Risk is Predicted**	Questionnaire and radiofrequency scan	Questionnaire, radiofrequency scan, and genetic test	
**How Many Appointments are Needed**	Two	One	You would not have your risk of breast cancer predicted
**Location of Appointment**	Hospital	Your GP	You would be invited to breast cancer screening at age 50
**Possible Times for Appointment**	Weekdays only	Weekdays, Evenings and Weekends	If you were worried about cancer

	**Risk Prediction 1**	**Risk Prediction 2**	**No Risk Prediction**
**How Appointments are Booked**	Book a time yourself online or on the phone	Book a time yourself online or on the phone	before this, you would visit your GP
**The likelihood you would be estimated to be at high risk**	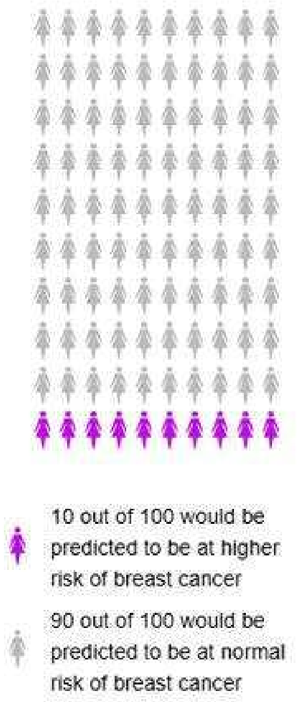	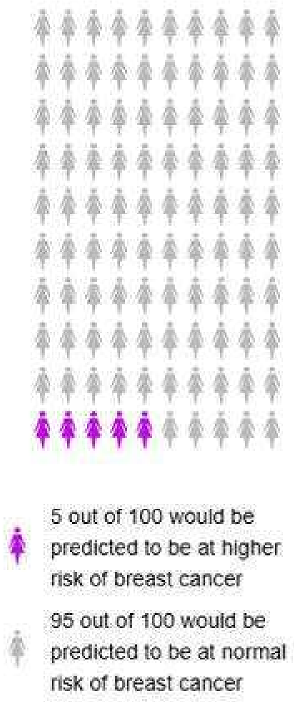	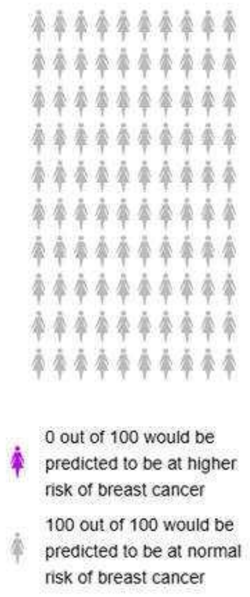

	Test 1	Test 2	No Risk Prediction
Your choice:	○	○	○

If you had to choose between the following breast cancer risk prediction services, which would you choose? You can also choose not to receive breast cancer risk prediction

	Risk Prediction 1	Risk Prediction 2	No Risk Prediction
**How Risk is Predicted**	Questionnaire and radiofrequency scan	Questionnaire	
**How Many Appointments are Needed**	Two	One	You would not have your risk of breast cancer predicted
**Location of Appointment**	Mobile Van	Hospital	You would be invited to breast cancer screening at age 50
**Possible Times for Appointment**	Weekdays, Evenings and Weekends	Weekdays, Evenings and Weekends	If you were worried about

	**Risk Prediction 1**	**Risk Prediction 2**	**No Risk Prediction**
			cancer before this, you would visit your GP
**How Appointments are Booked**	Receive a letter with a fixed time	Book a time yourself online or on the phone	
**The likelihood you would be estimated to be at high risk**	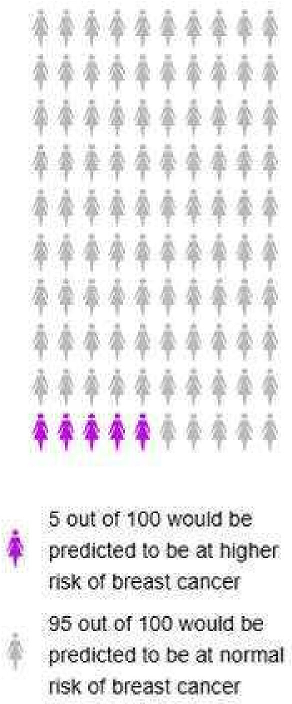	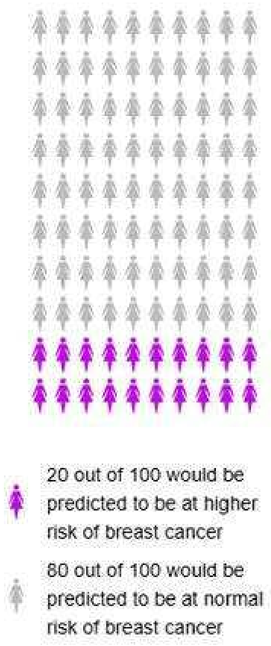	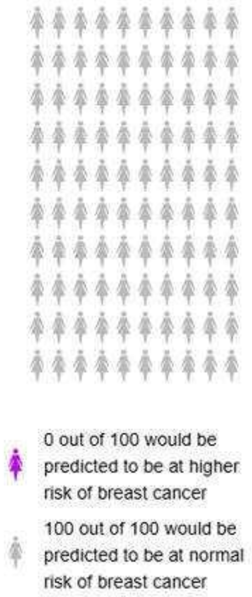

	Test 1	Test 2	No Risk Prediction
Your choice:	○	○	○

If you had to choose between the following breast cancer risk prediction services, which would you choose? You can also choose not to receive breast cancer risk prediction

	**Risk Prediction 1**	**Risk Prediction 2**	**No Risk Prediction**
**How Risk is Predicted**	Questionnaire, mammography scan and genetic test	Questionnaire, mammography scan and genetic test	
**How Many Appointments are Needed**	One	Two	You would not have your risk of breast cancer predicted
**Location of Appointment**	Hospital	Mobile Van	You would be invited tc breast cancer screening at age 50
**Possible Times for Appointment**	Weekdays, Evenings and Weekends	Weekdays, Evenings and Weekends	If you were worried about cancer

	Risk Prediction 1	Risk Prediction 2	No Risk Prediction
			before this, you would visit your GP
**How Appointments are Booked**	Receive a letter with a fixed time	Receive a letter with a fixed time	
**The likelihood you would be estimated to be at high risk**	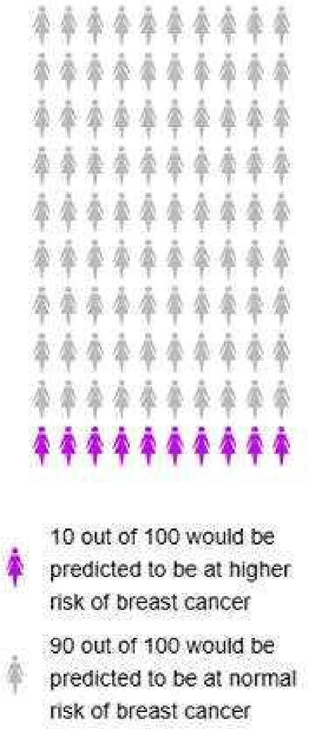	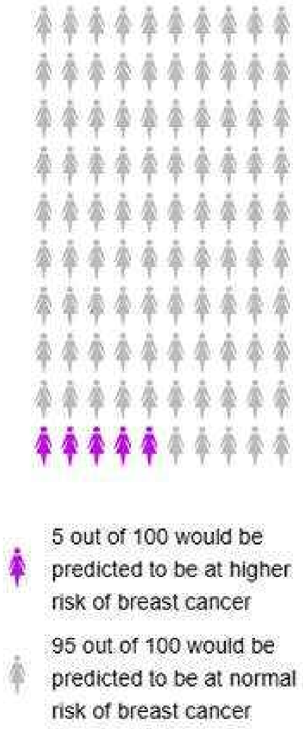	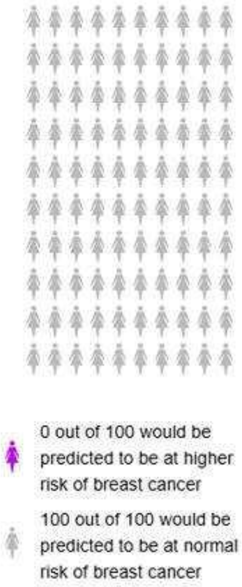

	Test 1	Test 2	No Risk Prediction
Your choice:	○	○	○

If you had to choose between the following breast cancer risk prediction services, which would you choose? You

can also choose not to receive breast cancer risk prediction

	Risk Prediction 1	Risk Prediction 2	No Risk Prediction
**How Risk is Predicted**	Questionnaire	Questionnaire, radiofrequency scan, and genetic test	
**How Many Appointments are Needed**	One	Two	You would not have your risk of breast cancer predicted
**Location of Appointment**	Community Centre	Your GP	You would be invited to breast cancer screening at age 50
**Possible Times for Appointment**	Weekdays only	Weekdays only	If you were worried about cancer before this,

	**Risk Prediction 1**	**Risk Prediction 2**	**No Risk Prediction**
**How Appointments are Booked**	Receive a letter with a fixed time	Book a time yourself online or on the phone	you would visit your GP
**The likelihood you would be estimated to be at high risk**	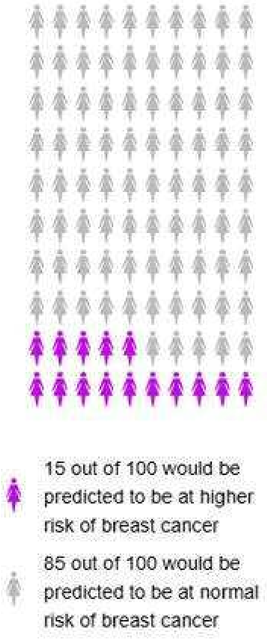	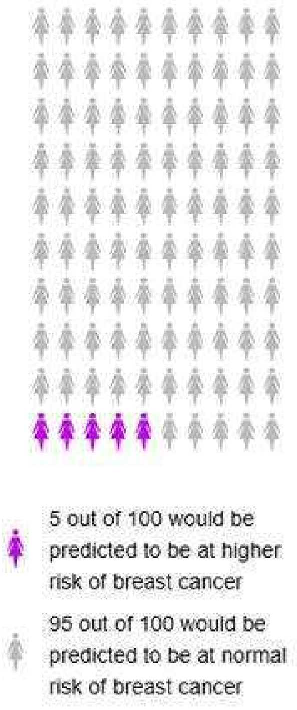	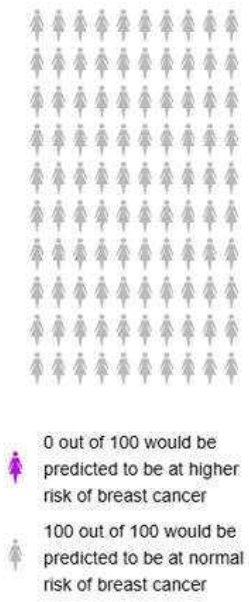

	Test 1	Test 2	No Risk Prediction
Your choice:	○	○	○

If you had to choose between the following breast cancer risk prediction services, which would you choose? You can also choose not to receive breast cancer risk prediction

	Risk Prediction 1	Risk Prediction 2	No Risk Prediction
**How Risk is Predicted**	Questionnaire, mammography scan and genetic test	Questionnaire and mammography scan	
**How Many Appointments are Needed**	One	One	You would not have your risk of breast cancer predicted
**Location of Appointment**	Mobile Van	Hospital	You would be invited tc breast cancer screening at age 50
**Possible Times for Appointment**	Weekdays only	Weekdays, Evenings and Weekends	If you were worried about cancer

	**Risk Prediction 1**	**Risk Prediction 2**	**No Risk Prediction**
			before this, you would visit your GP
**How Appointments are Booked**	Book a time yourself online or on the phone	Book a time yourself online or on the phone	
**The likelihood you would be estimated to be at high risk**	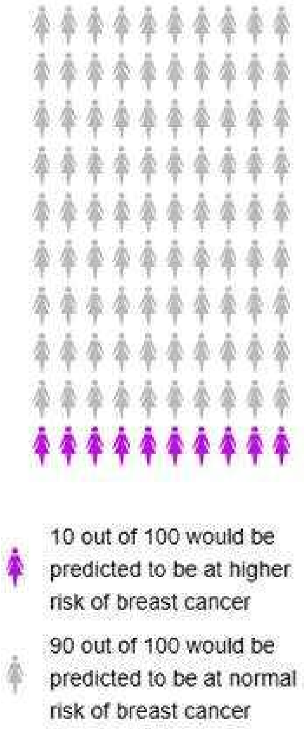	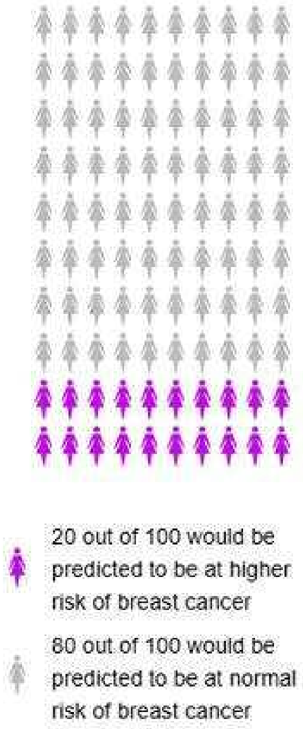	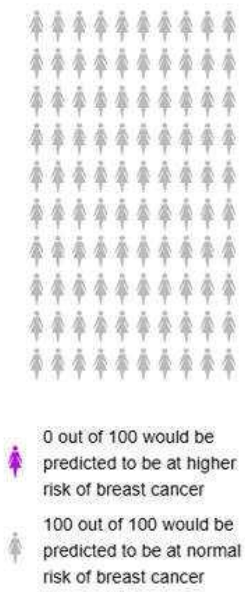

	Test 1	Test 2	No Risk Prediction
Your choice:	○	○	○

If you had to choose between the following breast cancer risk prediction services, which would you choose? You

can also choose not to receive breast cancer risk prediction

	Risk Prediction 1	Risk Prediction 2	No Risk Prediction
**How Risk is Predicted**	Questionnaire, radiofrequency scan, and genetic test	Questionnaire and genetic test	
**How Many Appointments are Needed**	One	One	You would not have your risk of breast cancer predicted
**Location of Appointment**	Mobile Van	Community Centre	You would be invited to breast cancer screening at age 50
**Possible Times for Appointment**	Weekdays only	Weekdays only	If you were worried about cancer before this,

<table>
<tr><th></th><th>Risk Prediction 1</th><th>Risk Prediction 2</th><th>No Risk Prediction</th></tr>
<tr><td>How Appointments are Booked</td><td>Receive a letter with a fixed time</td><td>Receive a letter with a fixed time</td><td>you would visit your GP</td></tr>
<tr><td>The likelihood you would be estimated to be at high risk</td><td>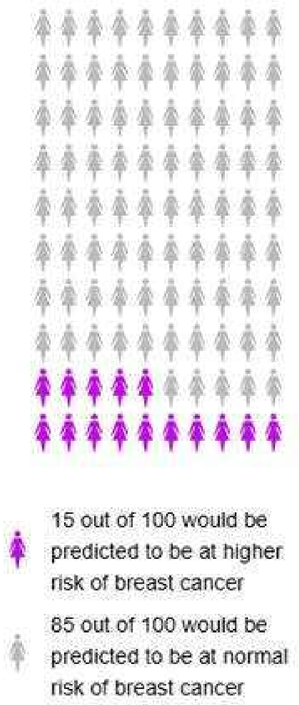
</td><td>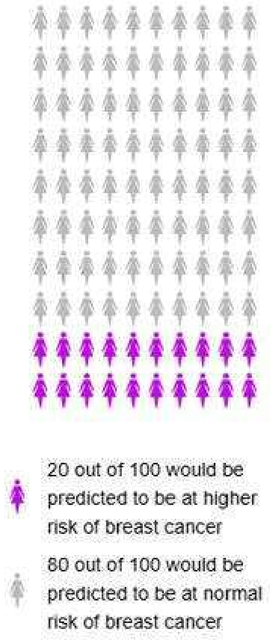
</td><td>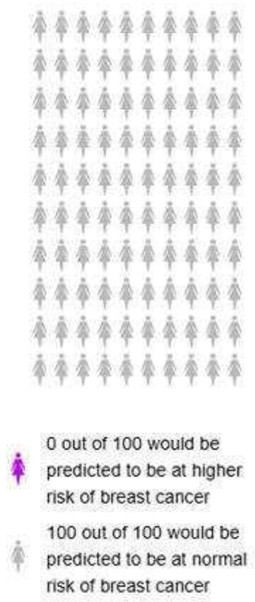
</td></tr>
</table>

	Test 1	Test 2	No Risk Prediction
Your choice:	○	○	○

dcecheck

Thanks for completing those questions.

We'll now ask you some questions about how you found completing that task

How easy or difficult did you find it to answer these questions?

Very difficult	Somewhat difficult	Neither easy nor difficult	Somewhat easy	Very easy
○	○	○	○	○

Did you use all of the characteristics of the service to make your choices or did you focus on just a few?

- ○ I always looked at all of the characteristics to decide
- ○ I looked at one or a few characteristics that were important to me
- ○ I never chose to have the risk prediction service

dcecheck2

Which of the characteristics did you use to make your choices? (select all that apply)

- ☐ How risk was predicted

- ☐ How many appointments would be needed
- ☐ Where you would need to go
- ☐ What time appointments are available
- ☐ How you can book an appointment
- ☐ The chance that your risk is overestimated
- ☐ The chance that your risk is underestimated

demographics

Think about the following statements in terms of how you react when you're dealing with health concerns

Please mark how much you agree with each statement

	Strongly disagree	Somewhat disagree	Neither agree nor disagree	Somewhat agree	Strongly agree
I like to gather as much information as I can before I make a decision	○	○	○	○	○
I have difficulty making sense of information from multiple sources	○	○	○	○	○
I fear that I might find out something I don't want to know	○	○	○	○	○
I like to review information multiple times before making a decision	○	○	○	○	○

	Strongly disagree	Somewhat disagree	Neither agree nor disagree	Somewhat agree	Strongly agree
I like to make decisions quickly	○	○	○	○	○
After I've made a decision, I continue to look for related information	○	○	○	○	○
I think it's the doctor's job to deal with information, not mine	○	○	○	○	○
I feel overwhelmed by the amount of information available	○	○	○	○	○

What is the highest level of education you have attained?

- ○ No formal qualifications
- ○ 1-4 O levels/GCSE's
- ○ 5+ O levels/GCSE's
- ○ National Vocational Qualifications (NVQs)
- ○ A levels/AS levels
- ○ Undergraduate degree
- ○ Master's degree
- ○ PhD/Doctorate
- ○ Other formal qualification

What is your religion?

- No religion
- Christian
- Buddhist
- Hindu
- Jewish
- Muslim
- Sikh
- ____ Other (please specify)

What is your ethnic group?

Choose one option that best describes your ethnic group or background

- White English/Welsh/Scottish/Northern Irish/British
- White Irish
- White Gypsy or Irish Traveller
- Other white background
- White and Black Carribbean
- White and Black African
- White and Asian
- Other mixed/multiple ethnic background
- Indian
- Pakistani
- Bangladeshi

- Chinese
- Other Asian background
- Black African
- Black Carribbean
- Any other Black/African/Caribbean background
- Arab
- Any other ethnic group

Do you have any children?

- Yes
- No

Are you generally a person who is fully prepared to take risks or do you try to avoid taking risks?

Please move the slider to indicate your position on the scale, where 0 means 'risk averse' and 10 means 'fully prepared to take risks.' You may choose any value between 0 and 10 that best represents your estimate.

Risk averse | Neutral | Fully prepared to take risks

0 1 2 3 4 5 6 7 8 9 10

Slide the slider

People can behave differently in different situations. How would you rate your willingness to take risks in the follow areas?

Please move the slider to indicate your position on the scale, where 0 means 'risk averse' and 10 means 'fully prepared to take risks.' You may choose any value between 0 and 10 that best represents your estimate.

	Risk averse					Neutral					Fully prepared to take risks	
	0	1	2	3	4	5	6	7	8	9	10	
while driving?												
in financial matters?												
during leisure and sport?												
in your occupation?												
with your health?												
your faith in other people												

Block 12

You have now completed the survey

Thank you for taking the time to complete this survey.

Additional information about breast cancer risk can be found at the following websites:
Cancer Research UK - Breast Cancer Risk
NHS - Breast Cancer in Women
Cancer Research UK - Reducing Your Risk of Breast Cancer

Please continue to be redirected to the panel provider website

Powered by Qualtrics
