# Supplementary material for: Understanding the preferences of younger women for the delivery of a service to predict breast cancer risk: a discrete choice experiment
Source: BJC Rep. 2026 Mar 13;4:10. doi: 10.1038/s44276-026-00209-x (PMC12987935; doi:10.1038/s44276-026-00209-x)
Supplement: Supplementary file 2 — Supplementary Appendix 2 [file 44276_2026_209_MOESM2_ESM.docx]

**Supplementary Appendix 2: Understanding the preferences of younger women for the delivery of a service to predict breast cancer risk: a discrete choice experiment**

**Short title:**  Preferences for a breast cancer risk-prediction service

Stuart J Wright^1^

Shabnam Thapa^2^

Amber Salisbury^1^

Sarah Hindmarch^3^

David P French^3^

Sacha J Howell^4^

Katherine Payne^1^

^1^Manchester Centre for Health Economics, Division of Population Health, Health Services Research and Primary Care, School of Health Sciences, Faculty of Biology, Medicine and Health, The University of Manchester, Manchester, United Kingdom.

^2^National Institute for Health and Care Excellence, Manchester, United Kingdom

^3^Manchester Centre for Health Psychology, Division of Psychology and Mental Health, School of Health Sciences, Faculty of Biology, Medicine and Health, The University of Manchester, Manchester, United Kingdom.

^4^Manchester Cancer Research Centre, Division of Cancer Sciences, School of Medical Sciences, Faculty of Biology, Medicine and Health, University of Manchester, Manchester, United Kingdom

**Corresponding author:** Stuart Wright: [stuart.j.wright@manchester.ac.uk](mailto:stuart.j.wright@manchester.ac.uk)

ORCID: [0000-0002-4064-7998](https://orcid.org/0000-0002-4064-7998)

**Contents:**

**Supplementary appendix 2.1:** Reporting Checklist for Discrete Choice Experiments in Health - The DIRECT Checklist

**Supplementary appendix 2.2:** Model fit statistics

**Supplementary appendix 2.3:** Standard deviations of Random Parameter Logit model with Pseudo Panel effects

**Supplementary Appendix 1.1:** Reporting Checklist for Discrete Choice Experiments in Health - The DIRECT Checklist

| Reporting criteria | | Page number (abstract a page 1) |
| --- | --- | --- |
| Purpose and rationale | |  |
| 1 | Describe the real-world context and decision-maker that the hypothetical choice context seeks to replicate or inform | Page 2, paragraph 1 |
| 2 | Provide a rationale for using a DCE to answer the research question | Page 2, paragraph 2 |
| Attributes and levels^a^ | |  |
| 3 | Describe how attributes and levels were derived (e.g. literature review, interviews, focus groups, expert input) | Page 3, paragraph |
| 4 | Provide the final list of attributes and levels | Page 3, paragraph 4, Table 1 |
| Experimental design | |  |
| 5 | Report the number of alternatives per choice set and whether they were labelled or unlabelled | Page 3, paragraph 4 |
| 6 | Describe response options (e.g. forced choice, opt-out, status quo) | Page 3, paragraph 4 |
| 7 | Describe the type of experimental design (e.g. orthogonal, D-efficient, Bayesian efficient, partial profile) | Page 4, paragraph 2 |
| 8 | Describe which effects are identified in the design (e.g. main effects, higher order interactions, functional form) | Page 4, paragraph 2 |
| 9 | Describe the number of choice sets, blocks and choice sets per block | Page 4, paragraph 2 |
| 10 | Indicate how the experimental design was obtained (software, catalogue, other) | Page 4, paragraph 2 |
| Survey design | |  |
| 11 | Provide a sample choice set and the instructions and background information given to respondents (e.g. providing the survey as an appendix) | Appendix 2 |
| 12 | Report any randomisation (e.g. choice set order, attribute order, alternative order, framing effects) | Page 4, paragraph 2 |
| 13 | Describe what was checked in piloting (e.g. understanding, respondent burden, timing, wording) | Page 4, paragraph 4 |
| 14 | Report whether information from the pilot was used to update the experimental design (e.g. priors, functional form of attributes) or survey design | Page 5 paragraph 1 |
| Sample and data collection | |  |
| 15 | Report respondent inclusion/exclusion criteria | Page 5, paragraph 1 |
| 16 | Describe how data were collected (e.g. mail, personal interview, web survey) | Page 5, paragraph 1 |
| 17 | Report the response rate or cooperation rate, if possible | Page 5, paragraph 6 |
| 18 | Report the final sample size and how the sample size was determined | Page 5, paragraph 2 |
| 19 | Describe respondent characteristics and representativeness of target population, if known | Page 9, paragraph 1 |
| Econometric analysis | |  |
| 20 | Indicate coding of data (e.g. effects, dummy, continuous) including definitions | Page 5, paragraph 4 |
| 21 | Report whether any respondents were removed and why (e.g. suspected fraudulent responses, rationality tests) | Page 5, paragraph 3 |
| 22 | Provide the rationale for model choice (e.g. conditional logit, mixed logit, latent class) and assumptions (e.g. error variance) | Page 6, paragraphs 6, 7 and 8 |
| 23 | Report model specification | Page 10, paragraph 2 |
| Reporting of results | |  |
| 24 | Report the model performance, goodness of fit (if comparing models) | Page 10, paragraph 2 |
| 25 | Describe methods used for analysis of model results (e.g. calculation of marginal rate of substitution, attribute relative importance, welfare gain) | Page 6, paragraph 5 |
| 26 | Report measures of precision for the output(s) of interest (e.g. confidence intervals) and how these were derived | Page 12, Table 4  Page 15, Table 5 |

**Source:** [**https://pmc.ncbi.nlm.nih.gov/articles/PMC11405421/**](https://pmc.ncbi.nlm.nih.gov/articles/PMC11405421/)

**Appendix 2.1**: Model fit statistics

|  | **Akaike Information Criterion** | **Bayesian Information Criterion** |
| --- | --- | --- |
| **Conditional logit (1 alternative specific constant)** | 18087 | 18187 |
| **Conditional logit (2 alternative specific constant)** | 18087 | 18194 |
| **Conditional logit (square term for the proportion at high risk attribute)** | 18087 | 18194 |
| **Conditional logit (logarithmic term for the proportion at high risk attribute)** | 18088 | 18195 |
| **Uncorrelated Random Parameter Logit** | 14628 | 14828 |
| **Uncorrelated Random Parameter Logit with Pseudo Panel Effects^1^** | 14607 | 14815 |
| **Uncorrelated Random Parameter Logit with Heteroscedasticity** | 14613 | 14820 |
| **Fully Correlated Random Parameter Logit** | 14428 | 15278 |
| **Latent Class (2 classes)** | 15394 | 15601 |
| **Latent Class (3 classes)** | 14963 | 15278 |
| **Latent Class (4 classes)^1^** | 14688 | 15110 |
| **Latent Class (5 classes)** | Did not converge | Did not converge |

^1^ Selected model for analysis

**Appendix 2.3:** Standard deviations of Random Parameter Logit model with Pseudo Panel effects

| **Attribute/Level** | **Estimate^1^** | **Robust standard error** | **Robust p-value** |  |
| --- | --- | --- | --- | --- |
| **Number of appointments** | 0.206 | 0.280 | 0.461 |  |
| **Appointments available at weekends** | -0.040 | 0.056 | 0.480 |  |
| **Can book appointments yourself** | 0.179** | 0.063 | 0.004 |  |
| **Location** | | | | |
| Hospital | 0.273* | 0.113 | 0.015 |  |
| Community centre | -0.433*** | 0.079 | <0.001 |  |
| Mobile Van | -0.141 | 0.101 | 0.165 |  |
| Home | -0.226 | 0.202 | 0.263 |  |
| Likelihood you found to be at high risk | 0.107*** | 0.008 | <0.001 |  |
| **Mode of Risk Prediction** | | | | |
| Questionnaire and genetic test | -0.075 | 0.145 | 0.607 |  |
| Questionnaire and mammography | 0.027 | 0.184 | 0.883 |  |
| Questionnaire, mammography, and genetic test | -0.825*** | 0.097 | <0.001 |  |
| Questionnaire and radiofrequency | -0.077 | 0.124 | 0.533 |  |
| Questionnaire, radiofrequency and genetic test | 0.741*** | 0.098 | <0.001 |  |
| **Alternative Specific Constant** | 4.022*** | 0.230 | <0.001 |  |

^1^ Where attributes and levels have statistically significant estimates, this suggests that there is evidence of heterogeneity in participant’s preferences for these attributes, regardless of the direction of the estimate.
